# Type-2 diabetes and risk of dementia: observational and Mendelian randomisation studies in 1 million individuals

**DOI:** 10.1017/S2045796020000347

**Published:** 2020-04-24

**Authors:** Jesper Qvist Thomassen, Janne Schurmann Tolstrup, Marianne Benn, Ruth Frikke-Schmidt

**Affiliations:** 1 Department of Clinical Biochemistry, Rigshospitalet, Copenhagen, Denmark; 2 National Institute of Public Health, University of Southern Denmark, Odense, Denmark; 3 Department of Clinical Medicine, Faculty of Health and Medical Sciences, University of Copenhagen, Copenhagen, Denmark

**Keywords:** Alzheimer's disease, dementia, Mendelian randomisation, type-2 diabetes, unspecified dementia, vascular dementia

## Abstract

**Aims:**

In observational studies, type-2 diabetes is associated with increased risk of dementia; however, the causal nature of this association remains unanswered. In an unselected nationwide study of all Danes, we wanted to test whether type-2 diabetes is associated with dementia subtypes, and to test whether potential associations are of a causal nature.

**Methods:**

In the current study of nationwide observational registry data in all Danes above the age of 65 years (*n* = 784 434) combined with genetic consortia data on 213 370 individuals, we investigated the associations between type-2 diabetes and Alzheimer's disease, vascular dementia, unspecified dementia and all-cause dementia, and whether observational associations were of a causal nature by applying a two-sample Mendelian randomisation strategy. We addressed key biases inherent in Mendelian randomisation approaches.

**Results:**

Important confounders (age, ethnicity, size of community, region, civil status and education level) were captured on all 784 434 individuals and adjusted for in the models. Multifactorial adjusted hazard ratios were 1.13 (1.06–1.21) for Alzheimer's disease, 1.98 (1.83–2.14) for vascular dementia, 1.53 (1.48–1.59) for unspecified dementia and 1.48 (1.44–1.53) for all-cause dementia in persons with type-2 diabetes *v*. without. Results were similar for men and women. The two-sample Mendelian randomisation estimate for the association between the genetic instrument and Alzheimer's disease was 1.04 (0.98–1.10), consistent with sensitivity estimates, addressing pleiotropy, measurement bias and weak instrument bias.

**Conclusions:**

In a nationwide study of all Danes above the age of 65 years, we show that type-2 diabetes is associated with major subtypes of dementia – with particularly strong associations for vascular dementia and unspecified dementia – the two types of dementia with the most obvious vascular pathologies. Although the present two-sample Mendelian randomisation approach using genetic consortia data suggests that type-2 diabetes is not a direct cause of Alzheimer's disease, we were unable to test the causal nature of type-2 diabetes for vascular dementia and unspecified dementia, because no publicly available genetic consortia data yet exist for these dementia endpoints. The causal nature of type-2 diabetes for dementia with vascular pathologies is pivotal questions to solve for future public health recommendations and therapeutic advice.

## Introduction

Due to ageing of populations, the number of people with dementia worldwide is anticipated to triple between 2015 and 2050 (Livingston *et al*., 2017). The global years of life lost due to dementia increased by 37.5% from 1990 to 2015, and dementia is now the fourth leading cause of death in high-income countries (Naghavi *et al*., 2017). Recent estimates suggest that up to a third of all dementia may be attributable to modifiable risk factors, among these type-2 diabetes (Livingston *et al*., 2017). The worldwide prevalence of type-2 diabetes has doubled since 1980 to 8.5% in 2014 (422 million people) reflecting that the prevalence of overweight and obesity continues to increase in all regions of the world (World Health Organization, 2016). Several large observational studies and meta-analyses have shown that type-2 diabetes is associated with higher risk of dementia (Price *et. al.*, 2014; Chatterjee *et al*., 2016). The relationship between dementia subtypes and the causal nature of this association remains however unanswered and is a central question to resolve for future public health recommendations and therapeutic advice and development.

Despite strong observational evidence for the association between type-2 diabetes and dementia, observational studies are prone to confounding and reverse causation, and therefore cannot establish causality (Katan, 1986; Smith and Ebrahim, 2003; Smith *et al*., 2005; Hernan and Robins, 2006). Mendelian randomisation is an epidemiological approach that aims to circumvent confounding and reverse causation using genetic variants in human populations. Because of random assortment of alleles at conception, genetic variants associated with a modifiable exposure are randomly distributed in relation to potential confounders (Smith and Ebrahim, 2003; Smith *et al*., 2005). Therefore, genetic variants that associate with type-2 diabetes can be used as unconfounded proxies to construct a genetic instrument to study the causal nature of the association between type-2 diabetes and risk of dementia. Furthermore, because germline variation is determined at gamete formation and conception and remains unchanged throughout life, Mendelian randomisation minimises the influence of reverse causation. Although Mendelian randomisation methods are not influenced by confounding and reverse causation, strong assumptions are made when the genetic instrument is constructed.

In the current study, we combine the use of a nationwide registry-based study in the Danish population with a two-sample Mendelian randomisation approach to evaluate associations between type-2 diabetes and dementia subtypes and to study the causal nature of potential associations. First, we used a nationwide registry-based study in the Danish population to test the association between type-2 diabetes and risk of Alzheimer's disease, vascular dementia, unspecified dementia and all-cause dementia. Second, we used publicly available consortia data to construct several instruments from genetic variants associated with type-2 diabetes. We investigated the validity of the genetic instruments using various methods and sensitivity analyses. Finally, we used two-sample instrumental variable analysis to test whether the association between type-2 diabetes and Alzheimer's disease is causal.

## Method

### Study populations and consortia data

#### Nationwide observational study

The Danish Civil Registration System records all births, immigrations, emigrations and deaths in Denmark by means of civil registration numbers, which uniquely identify all inhabitants in Denmark and include information regarding age, sex, ethnicity and civil status. The Danish Civil Registration System is 100% complete and for practical purposes, no persons are lost to follow-up (Pedersen *et al*., 2006). Studies were approved by the National Board of Health and the local Data Protection office.

#### Genetic consortia data

We downloaded summary estimates of genetic variants with effect on type-2 diabetes from the DIAbetes Genetics Replication And Meta-analysis (DIAGRAM) consortium (Scott *et al*., 2017) and summary estimates of genetic variants with effect on the risk of Alzheimer's disease from the International Genomics of Alzheimer's Project (IGAP) consortium (Lambert *et al*., 2013). The consortia are described in detail in the online Supplementary Methods section.

### Study design

**Fig. 1.**
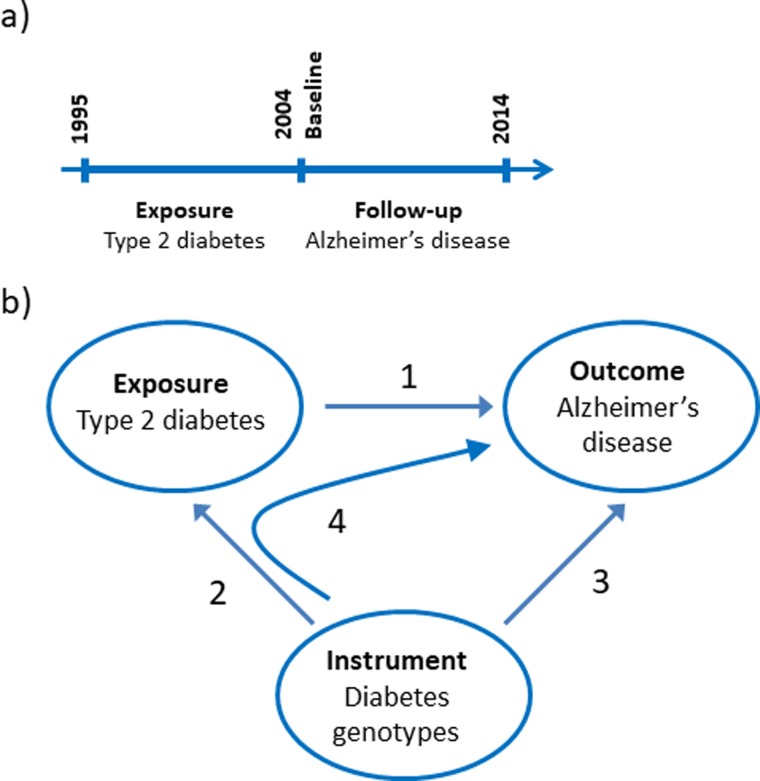
Study design. (*a*) Design of the observational study of the association between type-2 diabetes and Alzheimer's disease, vascular dementia, unspecified dementia and all-cause dementia. (*b*) Design of the Mendelian randomisation study: association of type-2 diabetes with Alzheimer's disease (1), construction of genetic instruments from diabetes associated genotypes (2), association of genetic instruments with Alzheimer's disease (3) and using instrumental variable analysis (4).

The nationwide prospective cohort study was designed using 1st of January 2004 as baseline where age and covariates were determined from registries (Fig. 1*a*). We limited the study to the population above 65 years of age at baseline and included 784 434 individuals. Type-2 diabetes was a registered diagnose code in the years 1995–2003. Dementia endpoints were determined in the period following the baseline from 2004 to 2014.

We used a two-sample Mendelian randomisation design to test the hypothesis whether type-2 diabetes causes Alzheimer's disease (Fig. 1*b*) (Burgess *et al*., 2015). First, we tested if a type-2 diabetes diagnosis is associated with higher risk of Alzheimer's disease using a nationwide prospective cohort study of the Danish population. Second, we constructed a genomic instrument where the individual genetic variants were associated with type-2 diabetes using the summarised data from DIAGRAM. Third, we calculated the causal estimate whether type-2 diabetes-affected risk of Alzheimer's disease using instrumental variable analysis. Last, we investigated whether a potential causal effect of type-2 diabetes was consistent with the corresponding observational association and evaluated the validity of the genetic instrument in sensitivity analyses.

### Diabetes diagnoses and dementia endpoints

In the nationwide observational study, information on diagnoses of Alzheimer's disease, vascular dementia, unspecified dementia and type-2 diabetes was collected from the Danish National Patient Registry with data on all patient contacts from all clinical hospital departments in Denmark since 1977 and including emergency wards and out-patient clinics from 1995. Time of deaths was obtained from the National Danish Causes of Death Registry with data on causes of all deaths in Denmark as reported by hospitals and general practitioners since 1977. Date of birth and time of emigration and/or immigration were obtained from the Danish Civil Registration System.

The diagnoses in the Danish National Patient Registry have been coded using World Health Organization International Classification of Disease and related health problems 10th revision (ICD10) from 1995. Type-2 diabetes was coded ICD10 as E11, E13 or E14, Alzheimer's disease F00 and G30, vascular dementia F01 and unspecified dementia F03. Furthermore, we constructed an all-cause dementia endpoint combining the codes from Alzheimer's disease, vascular dementia and unspecified dementia with censoring at the earliest date of one of the three diagnoses. The validity of dementia diagnoses in the Danish registries has previously been evaluated (Phung *et al*., 2011; Rasmussen, 2016). Follow-up ended at occurrence of event (dementia diagnosis), death, emigration or on 31st December 2014 whichever came first, and was 100% complete, that is, no individual was lost to follow- up. Median follow-up time was 9.9 years and total time-at-risk was 5 749 753 person-years. Individuals with events (diagnosed with dementia) before baseline were excluded from the observational study.

### Genetic instruments

Genetic variants associated with type-2 diabetes with *p*-values <10^−7^ were selected from the DIAGRAM dataset. The threshold of <10^−7^ was selected to ensure a high degree of independence of association between the genetic variants and type-2 diabetes even with multiple testing (Panagiotou *et al*., 2012). Pairwise linkage disequilibrium (LD) between genetic variants was determined using the SNiPA website (Arnold *et al*., 2015). Genetic variants with LD *r*^2^ ⩾ 0.8 were grouped and the genetic variant with the lowest *p* value in each group was selected for the genetic instrument. This procedure was repeated, constructing genetic instruments with pairwise *r*^2^ < 0.8, *r*^2^ < 0.6, *r*^2^ < 0.4 and *r*^2^ < 0.2, respectively. The genetic instruments are shown in online Supplementary Table 1.

Genetic variants directly affecting Alzheimer's disease are not valid in a genetic instrument, because the instrument is constructed to test the causal nature of a specific intermediate trait, in this case type-2 diabetes, for Alzheimer's disease. The apolipoprotein E gene (*APOE*) on chromosome 19 is strongly directly associated with Alzheimer's disease, hence all genetic variants near the *APOE* gene were excluded from all genetic instruments (MR-Egger plot with the *APOE* variants is shown in online Supplementary Fig. 1). Further, a fifth genetic instrument, referred to as the pathway instrument, was constructed including the genetic variants from the *r*^2^ < 0.2 instrument without any known biological associations that would disqualify the genetic variant – an example of a disqualifying gene is the highly pleiotropic *CDKN2A*/*B* gene (online Supplementary Table 1). The validity of a genetic instrument depends on whether any of the genetic variants included are associated with confounders or are directly affecting Alzheimer's disease. Therefore, we investigated whether the pathway instrument was associated with known confounders for type-2 diabetes and Alzheimer's disease using MR-Base (Gibran *et al*., 2018) with appropriate summary estimates from the Global Lipids Genetics Consortia (GLGC) (low-density lipoprotein (LDL) cholesterol, high-density lipoprotein (HDL) cholesterol and triglycerides), the Genetic Investigation of ANthropometric Traits (GIANT) (body mass index (BMI) and waist to hip ratio), the UK Biobank (BMI, systolic blood pressure, diastolic blood pressure, moderate physical activity, low physical activity and strenuous sport), the Social Science Genetic Association Consortium (SSGAC) (years of schooling) and finally for the causal association, the IGAP (Alzheimer's disease).

The instrument strength of each genetic variant was estimated using the formula for the *F*-statistics in (Burgess and Thompson, 2011). Sensitivity analyses were performed by restricting the pathway instrument to genetic variants with instrument strength >50, >75 and >100.

### Statistical analysis

Stata MP 14 and Stata SE 14 with the mrrobust package were used for the observational study and for the two-sample Mendelian randomisation study, respectively (Spiller *et al*., 2019). Because Alzheimer's disease diagnoses before age 65 often have other aetiologies than the common late onset form of dementia, we included people >65 years of age in the observational nationwide study. Cox regression models with age as timescale were used to calculate hazard ratios with 95% confidence intervals (CIs). Multivariable Cox models were adjusted for gender, ethnicity, civil status, highest level of education, size of community/residential area and region of living. Furthermore, we split the dataset into 2-year intervals to capture variation in dementia diagnosis practice. To test whether reverse causation affected our results, a sensitivity analysis was made where dementia diagnoses in the first 2 years following baseline were excluded from the analysis. The proportional hazard assumption was investigated by stratifying individually on the covariates (online Supplementary Table 2). No major violations were found.

To obtain robust causal estimates of the risk of diabetes on Alzheimer's disease using the summary genome-wide association study data, we performed regression analysis using inverse variance weighting and evaluated the validity, weak instrument bias and pleiotropic effects using Mendelian randomisation Egger regression (MR-Egger) (Bowden *et al*., 2015), weighted median (Bowden *et al*., 2016*a*, *b*) and modal estimates (Hartwig *et al*., 2017). Inverse variance weighted estimator assumes no pleiotropy and that each genetic variant fulfils the Mendelian randomisation assumptions. The MR-Egger estimator allows and adjusts for pleiotropy. We used the SIMulation EXtrapolation method (SIMEX) in the cases where MR-Egger regression showed NO Measurement Error (NOME) assumption violation. NOME violation is a type of regression dilution bias. Weighted median and modal estimators relaxed the assumption that all genetic variants in the instrument must be valid using different methods yielding consistent causal estimates even when more that 50% of the variants are invalid.

## Results

Baseline characteristics of the 784 434 individuals by gender combined and stratified by sex are shown in Table 1. Age at entry, ethnicity, size of community, region, civil status and education level are shown. Individuals with type-2 diabetes (5.1%) were more often men, had immigrant background, were widowed or divorced and high school or less as the highest education obtained compared to individuals without type-2 diabetes (95%).

**Table 1.** Characteristics of study participants in the observational study by type-2 diabetes status at baseline

	All		Women		Men	
	No type-2 diabetes	Type-2 diabetes	No type-2 diabetes	Type-2 diabetes	No type-2 diabetes	Type-2 diabetes
*N* (%)	744 440 (94.9%)	39 994 (5.1%)	429 700 (95.5%)	20 208 (4.5%)	314 740 (94.1%)	19 786 (5.9%)
Sex – women	57.7% (429 700)	50.5% (20 208)	–	–	–	–
Age at entry						
25%	69.1	69.7	69.6	70.6	68.6	68.9
Median	74.2	74.7	75.1	76.1	73.1	73.4
75%	80.4	80.6	81.6	82.1	78.9	78.8
Ethnicity						
Immigrant	3.5% (25 486)	5.5% (2100)	3.8% (16 228)	5.8% (1175)	2.9% (9257)	4.7% (379)
Descendant	0.1% (891)	0.1% (39)	0.1% (512)	0.1% (23)	0.1% (925)	0.1% (16)
Community size						
Greater Copenhagen	27.9% (207 603)	30.2% (12 067)	29.3% (126 055)	30.5% (6168)	25.9% (81 548)	29.8% (5899)
>50 000	7.7% (57 426)	9.1% (3651)	8.2% (35 106)	9.9% (1996)	7.1% (22 320)	8.4% (1655)
500– 50 000	50.2% (373 343)	48.4% (19 372)	50.9% (218 609)	49.1% (9923)	49.2% (154 733)	47.8% (9449)
<500	14.2% (106 069)	12.3% (4904)	11.6% (49 930)	10.5% (2121)	17.8% (56 139)	14.1% (2783)
Region						
Capital region	29.0% (215 974)	28.6% (11 443)	30.3% (130 211)	27.9% (5633)	27.2% (85 763)	29.4% (5810)
Middle region	21.1% (157 194)	20.1% (8058)	20.8% (89 339)	20.7% (4176)	21.6% (67 855)	19.6% (3882)
Southern region	22.9% (170 539)	24.2% (9680)	22.6% (97 020)	24.8% (5007)	23.4% (73 519)	23.6% (4673)
Zealand region	15.4% (114 475)	15.8% (6310)	15.0% (64 512)	15.3% (3096)	15.9% (49 963)	16.2% (3214)
Northern region	11.6% (86 258)	11.3% (4503)	11.3% (48 618)	11.4% (2296)	12.0% (37 640)	11.2% (2207)
Civil status						
Married	51.3% (381 698)	47.3% (18 904)	38.8% (166 733)	32.1% (6493)	68.3% (214 965)	62.7% (12 411)
Single	5.6% (41 973)	6.3% (2521)	5.2% (22 171)	4.7% (948)	6.3% (19 802)	8.0% (1573)
Widowed	33.7% (250 702)	35.0% (13 997)	46.1% (198 247)	51.6% (10 424)	16.7% (52 455)	18.1% (3573)
Divorced	9.4% (70 067)	11.4% (4572)	9.9% (42 549)	11.6% (2343)	8.7% (27 518)	11.3% (2229)
Highest education						
High school or less	45.7% (340 165)	49.1% (19 646)	50.2% (215 850)	55.0% (11 108)	39.5% (124 315)	43.2% (8538)
Short higher education	25.1% (187 027)	23.4% (9363)	19.8% (84 979)	15.4% (3121)	32.4% (102 048)	31.5% (6242)
Bachelor	8.6% (63 698)	6.1% (2452)	8.1% (34 624)	4.7% (944)	9.2% (29 074)	7.6% (1508)
Master or PhD	2.7% (19 767)	1.9% (748)	1.0% (4190)	0.4% (89)	4.9% (15 577)	3.3% (659)
Before register start	16.1% (120 202)	16.0% (6381)	19.3% (83 083)	20.8% (4198)	11.8% (37 119)	11.0% (2183)
Unknown	1.8% (13 581)	3.5% (1404)	1.6% (6974)	3.7% (748)	2.1% (6607)	3.3% (656)

Values are percent (number). Region refers to the five administrative regions in which the healthcare system in Denmark is divided into.

### Type-2 diabetes and risk of dementia: observational estimates

**Fig. 2.**
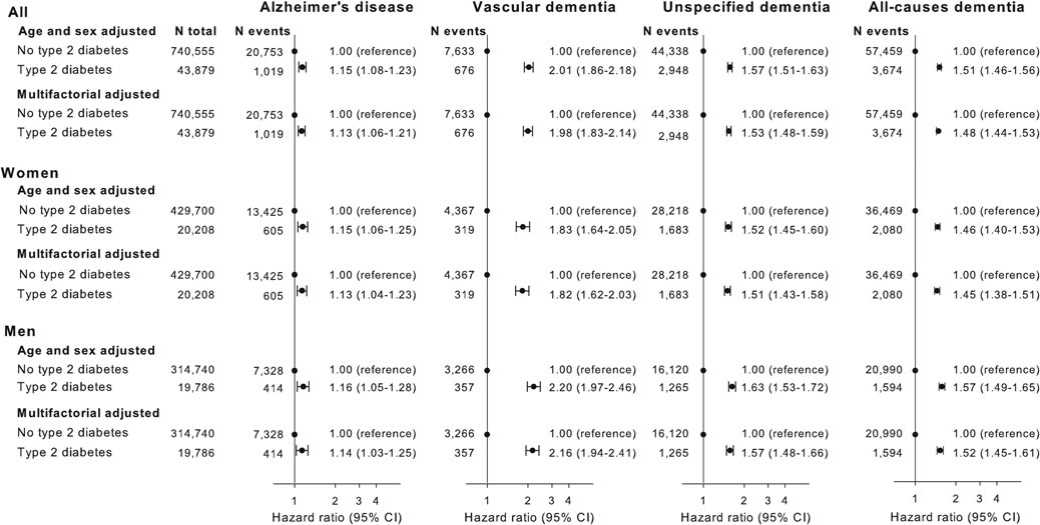
Result of observational study. Risk of Alzheimer's disease, vascular dementia, unspecified dementia and all-cause dementia as a function of type-2 diabetes status for both genders and separately adjusted for age, gender, educational level, ethnicity, community size, region and civil status.

Multifactorial adjusted hazard ratios were 1.13 (95% CI: 1.06–1.21) for Alzheimer's disease, 1.98 (1.83–2.14) for vascular dementia, 1.53 (1.48–1.59) for unspecified dementia and 1.48 (1.44–1.53) for all-cause dementia in persons with type-2 diabetes *v*. without (Fig. 2). Results were similar for men and women.

### Causal effect of type-2 diabetes on risk of dementia

**Fig. 3.**
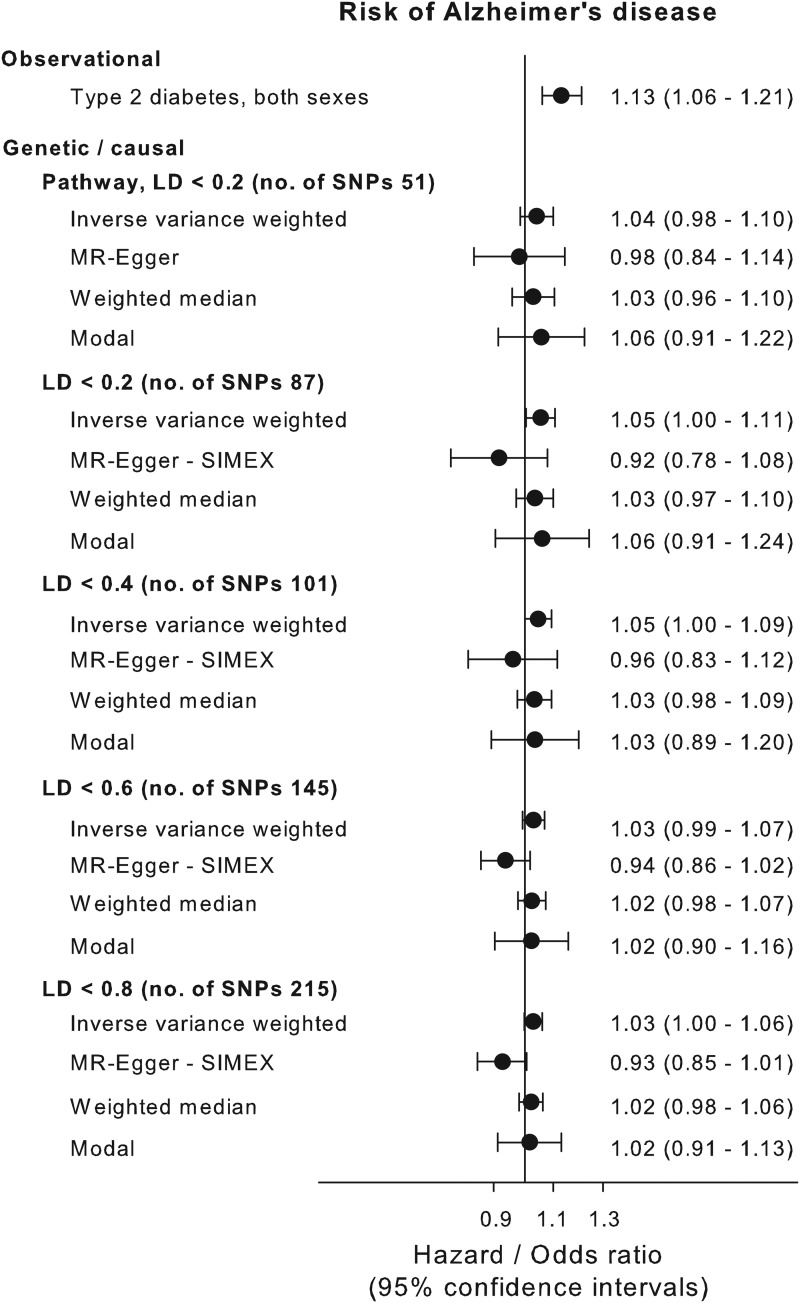
Causal estimates. Causal and observational risk estimates of Alzheimer's disease for type-2 diabetes. Causal risk was estimated using summary risk estimates from IGAP on genetic variants tested in DIAGRAM using conventional inverse variance weighted Mendelian randomisation analysis, Egger-Mendelian randomisation (MR-Egger), weighted median Mendelian randomisation and modal-based Mendelian randomisation for each of the five genetic instruments. Where needed the MR-Egger SIMEX estimation is used because of NOME bias (see online Supplementary Fig. 3). NOME = NO measurement error; SIMEX = SIMulation EXtrapolation method. Abbreviations for consortia are explained in the ‘Method’ section.

We applied the inverse variance weighted method for estimating causal inferences on five different genetic instruments based on the biological pathway and/or LD level and applied three different sensitivity methods to examine the validity of the genetic instruments. The genetic instruments are shown in online Supplementary Table 1. The instruments based solely on LD levels all showed violation of the NOME assumption (NOME estimate below 90%) according to MR-Egger regression (online Supplementary Fig. 2, right panel). MR-Egger with SIMEX was used to counter the NOME-violation and showed that LD < 0.8 and LD < 0.6 instrument had a weak pleiotropy, whereas the LD < 0.4 and LD < 0.2 did not show signs of pleiotropy (online Supplementary Fig. 2, middle panel). The pathway instrument showed no pleiotropy or NOME violation using the MR-Egger regression. MR-Egger plots and funnel plots are shown for each instrument in online Supplementary Fig. 3. The inverse variance weighted estimate for the association between the pathway instrument (variants with pairwise LD < 0.2 and without any known association with potential confounders, Alzheimer's disease and type-1 diabetes) and Alzheimer's disease was 1.04 (0.98–1.10) and was consistent with three sensitivity estimates, 0.98 (0.84–1.14), 1.03 (0.96–1.10) and 1.06 (0.91–1.22) for the MR-Egger, median weighted and modal methods, respectively (Fig. 3). The individual variants in the pathway instrument are shown as a forest plot in online Supplementary Fig. 4. Restricting the pathway instrument to variants with an *F*-statistics >50, >75 and >100 pulled the association towards the null hypothesis with greater instrument strength (online Supplementary Fig. 5). Results for instruments based solely on LD levels were largely similar to those observed for the pathway instrument (Fig. 3).

**Fig. 4.**
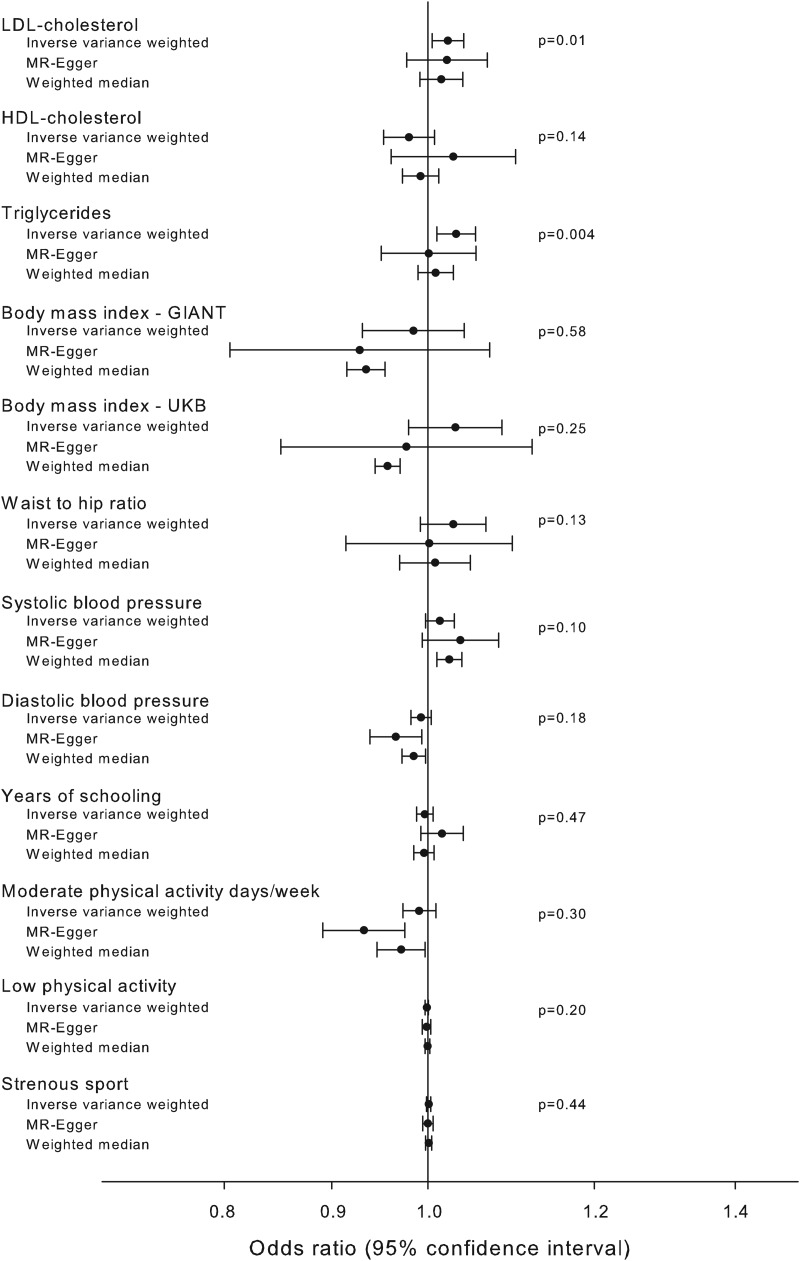
Association of pathway instrument with possible confounders. Estimated association of the pathway instrument with possible confounders of Alzheimer's disease and diabetes. The estimators using inverse variance weighted, MR-Egger and median weighted methods are shown. Summary estimates from GLGC (LDL cholesterol, HDL cholesterol and triglycerides), GIANT (BMI and waist to hip ratio), UK Biobank (BMI, systolic blood pressure, diastolic blood pressure, moderate physical activity, low physical activity and strenuous sport), SSGAC (years of schooling) and IGAP (Alzheimer's disease) consortia. Abbreviations for consortia are explained in the ‘Method’ section.

Associations between the pathway instrument and possible known confounders were investigated using MR-base (Gibran *et al*., 2018) (Fig. 4). The pathway instrument was weakly associated with higher concentrations of triglycerides (*β* = 0.03, *p* value = 0.004), and LDL cholesterol (*β* = 0.02, *p* value = 0.01) according to the inverse variance weighted method, however with *p* values not meeting a Bonferroni corrected level of significance and with non-significant results from MR-Egger and weighted median methods. All other tested confounders were not associated with the pathway instrument (Fig. 4).

## Discussion

In 784 434 citizens above 65 years of age from the Danish general population, we found that type-2 diabetes was associated observationally with high risk of dementia subtypes with particularly strong associations for vascular disease and unspecified dementia. Type-2 diabetes due to genetic variation was not associated with the risk of Alzheimer's disease when applying a two-sample Mendelian randomisation study of the DIAGRAM and IGAP consortia including 213 370 individuals. The null finding between genetically determined type-2 diabetes and Alzheimer's disease was consistent through various sensitivity analyses testing for pleiotropy, week instrument bias and no-measurement-error bias, as well as after controlling for potential confounding of the genetic instruments by including genetic lipid, BMI and hypertension consortia of >500 000 individuals.

To the best of our knowledge, a scrutinisation of potential pleiotropy-, weak or invalid instruments, and no-measurement-error bias has not been done previously in a two-sample-Mendelian randomisation study investigating a potential causal relationship between type-2 diabetes and Alzheimer's disease. Two previous Mendelian randomisation studies used older versions of DIAGRAM data from 2012. These studies neither investigated pleiotropy, nor validity of instruments or other potential sources of bias (Oestergaard *et al*., 2015; Larsson *et al*., 2017). Observational studies report risk estimates between 1.40 and 1.56 for type-2 diabetes in predicting Alzheimer's disease (Gudala *et al*., 2013; Price *et al*., 2014; Chatterjee *et al*., 2016). However, the size of the observational studies varies, and the different studies apply versatile schemes for adjusting for covariates. In contrast to our data, none of the studies were nationwide and were therefore prone to selection bias. Furthermore, the studies lack adjustment for potential confounders such as achieved educational level, civil status and demographic factors.

In two-sample Mendelian randomisation studies, a set of assumptions concerning the genetic instrument needs to be fulfilled to make valid conclusions about causality. Fortunately, we have methods that inform us of unbalanced pleiotropy and genetic heterogeneity assessed as NOME bias (MR-Egger method), weak instruments (*F*-statistics) and invalid instruments (median weighted and modal methods). Using these methods, we have demonstrated that we can remove unbalanced pleiotropy and NOME bias by restricting our genetic instrument to LD < 0.2 and removing potentially invalid instruments because of direct association with Alzheimer's disease. Furthermore, the robustness of the results when comparing with weighted median and modal methods indicate that we do have a valid instrument. The associations of the pathway instrument with concentrations of triglycerides and LDL cholesterol might invalidate the instrument if these lipid traits are causally associated with Alzheimer's disease. This has previously been investigated using two-sample Mendelian randomisation and no evidence of causality was observed (Oestergaard *et al*., 2015; Benn *et al*., 2017; Larsson *et al*., 2017). Furthermore, the associations with concentrations of triglycerides and LDL cholesterol were small and did not meet statistical significance after Bonferroni correction. Finally, by restricting the analysis to variants with an *F*-statistics above 100, we show that the strongest individual variants, that all are well known type-2 diabetes variants, pull the association with Alzheimer's disease further towards the null.

Type-2 diabetes is known to cause both macrovascular and microvascular complications and has been linked to brain atrophy likely caused by microaneurysms and/or microhaemorrhages arising due to hyperglycaemia (Barrett *et al*., 2017). Whether brain atrophy directly causes or is responsible for a more rapid progression of dementia is a matter of debate (Barrett *et al*., 2017). We found particularly strong observational associations for vascular dementia and unspecified dementia, suggesting involvement of macro- and/or microvascular pathologies. This may indicate a potential causal role of type-2 diabetes on the risk of dementias with the most obvious vascular pathologies. Whereas the present two-sample Mendelian randomisation suggests that type-2 diabetes is not a direct cause of Alzheimer's disease, we were unable to test the causal nature of type-2 diabetes for vascular dementia and unspecified dementia, because no publicly available genetic consortia data yet exists for these dementia endpoints.

Strengths of the study include examination of the entire Danish population above 65 years of age with access to individual citizen data of high validity and no losses to follow-up. The observational national data are unique due to a total lack of selection bias, since diagnoses from all hospital contacts, as well as all socioeconomic data are compiled in the national registries. The use of a two-sample Mendelian randomisation design, including a series of sensitivity analyses ensuring the validity of the genetic instrument, allowed us to examine potential causal effects of type-2 diabetes on Alzheimer's disease, largely without confounding and reverse causation. Furthermore, the genetic instrument was based on the DIAGRAM 2017 data which has a higher coverage of genetic variants throughout the genome compared with the previously used DIAGRAM 2012 data (Oestergaard *et al*., 2015; Larsson *et al*., 2017).

A limitation of the study is that the analysis is based on clinical diagnoses of dementia, as reflected in clinical practice. This is an important point, since discrepancies between clinical and neuropathological diagnoses of dementia and the relationship with type-2 diabetes have been observed (Sutherland *et al*., 2017). A recent study compared clinical diagnoses and pathological autopsy findings and concluded that Alzheimer's disease and vascular dementia display different profiles of organ and vascular damage as well as prevalence of concomitant hypertension and diabetes. Diabetes and hypertension were overrepresented in vascular dementia, whereas the prevalence of diabetes in Alzheimer's disease was comparable to that in the general population (Javanshiri *et al*., 2018). A body of neuropathological evidence shows that key features of Alzheimer's disease, such as extracellular deposits of amyloid *β* and intraneuronal aggregates of hyperphosphorylated tau, are not more frequent in patients with type-2 diabetes compared to individuals without (Arvanitakis *et al*., 2006; Abner *et al*., 2016; Pruzin *et al*., 2018). This is also supported by the fact that type-2 diabetes is not associated with cerebrospinal or positron emission tomography biomarkers of increased amyloid *β* accumulation (Moran *et al*., 2015). Taken together, the present findings suggesting that type-2 diabetes is not causally associated with Alzheimer's disease, are in line with studies on neuropathological evidence (Arvanitakis *et al*., 2006; Abner *et al*., 2016; Sutherland *et al*., 2017; Javanshiri *et al*., 2018; Pruzin *et al*., 2018). Hence, the present clinical diagnoses are probably appropriate to use for the current study. This is further supported by major efforts in Denmark since the mid-90s, in standardising dementia diagnoses according to guidelines.

In conclusion, we showed in an unselected nationwide study of all Danes above 65 years of age that type-2 diabetes is associated with major subtypes of dementia – with particularly strong associations for vascular dementia and unspecified dementia – the two types of dementia with the most obvious vascular pathologies. Whereas the present two-sample Mendelian randomisation approach using genetic consortia data suggests that type-2 diabetes is not a direct cause of Alzheimer's disease, we were unable to test the causal nature of type-2 diabetes for vascular dementia and unspecified dementia, because no publicly available genetic consortia data yet exist for these dementia endpoints. The causal nature of type-2 diabetes for dementia with vascular pathologies is pivotal questions to solve for future public health recommendations and therapeutic advice.

## Supplementary material

For supplementary material accompanying this paper visit http://dx.doi.org/10.1017/S2045796020000347. click here to view supplementary material

## Data

The data used for the nationwide observational study are available from Statistics Denmark for authorised researchers. The data used for Mendelian randomisation studies are available from the respective consortia's webpages: IGAP: web.pasteur-lille.fr/en/recherche/u744/igap/igap_download.php; DIAGRAM: www.diagram-consortium.org.
