# Supplementary material for: Type-2 diabetes and risk of dementia: observational and Mendelian randomisation studies in 1 million individuals
Source: Epidemiol Psychiatr Sci. 2020 Apr 24;29:e118. doi: 10.1017/S2045796020000347 (PMC7214711; doi:10.1017/S2045796020000347)
Supplement: Supplementary file 1 [file S2045796020000347sup001.docx]

**Type 2 diabetes and risk of dementia: observational and Mendelian randomization studies in 1 million individuals.**

**Supplementary Material**

JQ Thomassen MSc^1^, JS Tolstrup MSc, DMSc, PhD^2^, M Benn MD, DMSc, PhD^1,3^, R Frikke-Schmidt MD, DMSc, PhD^1,3^.

Supplementary Methods

| Supplementary Table 1: | Overview of genetic instruments. |
| --- | --- |
| Supplementary Table 2: | Stratified Cox sensitivity analysis to check proportionate hazards assumption. |
| Supplementary Figure 1: | MR-Egger plot with *APOE* SNPs. |
| Supplementary Figure 2: | MR-Egger sensitivity analysis. |
| Supplementary Figure 3: | MR-Egger plots with different genetic instruments. |
| Supplementary Figure 4: | Forrest plot of individual variants in pathway instrument. |
| Supplementary Figure 5: | Causal estimate dependence on strength of instrument. |

**Supplementary Methods**

**Genetic consortia data**

The DIAbetes Genetics Replication And Meta-analysis (DIAGRAM) consortium is an international collaboration with the purpose to elucidate the genetic structure of type 2 diabetes. We used data from DIAGRAM 1000G GWAS meta-analysis stage 1 Summary statistics dataset including 26,676 type 2 diabetes cases and 132,532 control subjects of European ancestry. Data from the 1000 Genomes project was used for genotype imputing and resulted in 12.3 M single nucleotide polymorphisms (SNPs) for association testing. This study provides a near-complete evaluation of common variants and improved coverage of low-frequency variants compared to previous studies (11).

The International Genomics of Alzheimer’s Project (IGAP) consortium is a large two-stage study based on genome-wide association studies of individuals of European ancestry. We used data from stage 1 including 7,055,881 genotyped and imputed SNPs to meta-analyze four previously published genome-wide association study datasets consisting of 17,008 Alzheimer’s disease cases and 37,154 controls (12).

**Covariates**

Since 1980, Statistics Denmark has gathered information concerning all persons living in Denmark. For this study, we obtained data on age, date of birth, sex, ethnicity, civil status, highest level of education, the size of community/residential area and the region individuals lived in at baseline.

|  |  |
| --- | --- |

**Supplementary Table 1: Overview of genetic instruments.**

|  |  |  |  |  | **DIAbetes Genetics Replication And Meta-analysis (DIAGRAM)** | | | | | | **International Genomics of Alzheimer's Project (IGAP)** | | | | **Genetic instrument** | | | | | | | | | |
| --- | --- | --- | --- | --- | --- | --- | --- | --- | --- | --- | --- | --- | --- | --- | --- | --- | --- | --- | --- | --- | --- | --- | --- | --- |
|  |  |  |  |  | Association with type 2 diabetes | | | | | | Risk of Alzheimer's disease | | | |  | |  | |  | |  | |  | |
| **SNP ID** | **Chr** | **POSITION** | **Associated gene** | **MAF (1000G CEU)** | **Effect allele** | **Non allele** | **β** | **SE** | **P-value** | **β** | | **SE** | **P-value** | **LD < 0.8** | | **LD < 0.6** | | **LD < 0.4** | | **LD < 0.2** | | **LD < 0.2 pathway** | |  |
| rs340874 | 1 | 214159256 | PROX1 | 0.47 | C | T | 0.068 | 0.012 | 3.4E-08 | -0.037 | | 0.016 | 2.13E-02 | X | | X | | X | | X | | X | |  |
| rs4846569 | 1 | 219771721 |  | 0.15 | C | T | 0.077 | 0.013 | 8.8E-09 | -0.036 | | 0.018 | 4.88E-02 | X | | X | | X | | X | |  | |  |
| rs7603255 | 2 | 43494177 | THADA | 0.12 | T | C | 0.110 | 0.019 | 5.5E-08 | 0.059 | | 0.024 | 1.52E-02 | X | | X | |  | |  | |  | |  |
| rs181122244 | 2 | 43658762 | THADA | 0.02 | G | A | 0.120 | 0.021 | 9.3E-09 |  | |  |  |  | |  | |  | |  | |  | |  |
| rs6712528 | 2 | 43707963 | THADA | 0.05 | G | A | 0.110 | 0.021 | 5.1E-08 |  | |  |  |  | |  | |  | |  | |  | |  |
| rs6757251 | 2 | 43734847 | THADA | 0.10 | C | T | 0.130 | 0.021 | 1.9E-10 | 0.060 | | 0.025 | 1.88E-02 | X | | X | | X | | X | | X | |  |
| rs181680672 | 2 | 43742503 | THADA | 0.002 | A | T | 0.130 | 0.024 | 3.1E-08 |  | |  |  |  | |  | |  | |  | |  | |  |
| rs6722104 | 2 | 43752346 | THADA | 0.09 | A | G | 0.130 | 0.021 | 3.0E-10 | 0.060 | | 0.025 | 1.88E-02 | X | | X | | X | | X | |  | |  |
| rs10193447 | 2 | 60552476 | BCL11A | 0.32 | T | C | 0.071 | 0.012 | 1.3E-08 | 0.006 | | 0.016 | 7.24E-01 | X | | X | | X | | X | | X | |  |
| rs968919 | 2 | 60579988 | BCL11A | 0.42 | C | T | 0.069 | 0.012 | 1.7E-08 | -0.021 | | 0.016 | 1.84E-01 | X | |  | |  | |  | |  | |  |
| rs243024 | 2 | 60583665 | BCL11A | 0.49 | A | G | 0.066 | 0.012 | 3.9E-08 | -0.007 | | 0.016 | 6.40E-01 | X | |  | |  | |  | |  | |  |
| rs13012438 | 2 | 60584283 | BCL11A | 0.28 | C | A | 0.068 | 0.013 | 7.6E-08 | -0.008 | | 0.017 | 6.27E-01 | X | |  | |  | |  | |  | |  |
| rs2972156 | 2 | 227117778 | IRS1 | 0.53 | G | C | 0.076 | 0.013 | 1.2E-09 | 0.014 | | 0.016 | 3.75E-01 | X | | X | | X | | X | | X | |  |
| rs6436615 | 2 | 227167075 | IRS1 | 0.33 | T | C | 0.073 | 0.013 | 4.2E-08 | 0.004 | | 0.018 | 8.37E-01 | X | | X | |  | |  | |  | |  |
| rs71304093 | 3 | 12275320 | PPARG | 0.04 | C | T | 0.160 | 0.026 | 2.0E-10 | -0.011 | | 0.033 | 7.27E-01 | X | | X | |  | |  | |  | |  |
| rs11715261 | 3 | 12317232 | PPARG | 0.05 | G | C | 0.140 | 0.024 | 2.8E-09 | -0.007 | | 0.030 | 8.17E-01 | X | |  | |  | |  | |  | |  |
| rs11712037 | 3 | 12344730 | PPARG | 0.09 | C | G | 0.130 | 0.019 | 8.6E-13 | 0.033 | | 0.024 | 1.62E-01 | X | | X | | X | | X | | X | |  |
| rs2881479 | 3 | 12367105 | PPARG | 0.12 | A | T | 0.094 | 0.017 | 4.1E-08 | 0.018 | | 0.022 | 4.26E-01 | X | | X | | X | | X | |  | |  |
| rs35352848 | 3 | 23455582 | UBE2E2 | 0.21 | T | C | 0.083 | 0.015 | 1.5E-08 | -0.003 | | 0.019 | 8.92E-01 | X | | X | | X | | X | | X | |  |
| rs7428936 | 3 | 64710850 | ADAMTS9 | 0.56 | T | C | 0.070 | 0.012 | 1.0E-08 | 0.021 | | 0.016 | 1.78E-01 | X | | X | | X | | X | |  | |  |
| rs11708067 | 3 | 123065778 | ADCY5 | 0.20 | A | G | 0.110 | 0.015 | 8.8E-13 | 0.035 | | 0.019 | 6.13E-02 | X | | X | | X | | X | | X | |  |
| rs11707746 | 3 | 123068817 | ADCY6 | 0.25 | T | C | 0.091 | 0.014 | 1.7E-10 | 0.025 | | 0.018 | 1.75E-01 | X | |  | |  | |  | |  | |  |
| rs57158761 | 3 | 185371172 | IGF2BP2 | 0.40 | G | A | 0.070 | 0.013 | 6.4E-08 | 0.008 | | 0.016 | 6.30E-01 | X | | X | | X | | X | | X | |  |
| rs6769946 | 3 | 185468884 | IGF2BP2 | 0.19 | T | C | 0.085 | 0.015 | 2.1E-08 | 0.008 | | 0.020 | 6.79E-01 | X | | X | | X | | X | | X | |  |
| rs10513800 | 3 | 185480388 | IGF2BP2 | 0.24 | A | C | 0.090 | 0.015 | 1.5E-09 | 0.007 | | 0.019 | 7.33E-01 | X | | X | |  | |  | |  | |  |
| rs71320320 | 3 | 185487155 | IGF2BP2 | 0.26 | T | A | 0.099 | 0.013 | 1.3E-13 |  | |  |  |  | |  | |  | |  | |  | |  |
| rs4402960 | 3 | 185511687 | IGF2BP2 | 0.33 | T | G | 0.140 | 0.013 | 2.7E-25 | 0.014 | | 0.017 | 3.98E-01 | X | | X | | X | | X | | X | |  |
| rs13100823 | 3 | 185514088 | IGF2BP2 | 0.30 | T | C | 0.130 | 0.013 | 1.2E-22 | 0.020 | | 0.017 | 2.52E-01 | X | | X | | X | | X | | X | |  |
| rs764129 | 3 | 185547917 | IGF2BP2 | 0.45 | T | C | 0.084 | 0.013 | 3.9E-11 | 0.012 | | 0.016 | 4.76E-01 | X | | X | |  | |  | |  | |  |
| rs77494444 | 3 | 185550500 |  | 0.06 | T | C | 0.180 | 0.030 | 2.0E-09 | 0.027 | | 0.052 | 6.00E-01 | X | | X | | X | | X | |  | |  |
| rs4996963 | 4 | 6276126 | WFS1 | 0.41 | A | G | 0.091 | 0.014 | 2.8E-11 | 0.035 | | 0.019 | 6.82E-02 | X | |  | |  | |  | |  | |  |
| rs3821940 | 4 | 6286711 | WFS1 | 0.57 | A | C | 0.089 | 0.012 | 4.4E-13 | 0.029 | | 0.016 | 6.83E-02 | X | | X | | X | | X | | X | |  |
| rs3821941 | 4 | 6286954 | WFS1 | 0.55 | G | A | 0.089 | 0.012 | 6.2E-13 | 0.030 | | 0.016 | 5.82E-02 | X | | X | | X | | X | | X | |  |
| rs1801213 | 4 | 6293696 | WFS1 | 0.63 | G | C | 0.083 | 0.013 | 1.0E-10 | 0.017 | | 0.017 | 3.31E-01 | X | | X | | X | | X | | X | |  |
| rs3821943 | 4 | 6299940 | WFS1 | 0.58 | T | C | 0.100 | 0.012 | 4.2E-16 | 0.028 | | 0.016 | 7.62E-02 | X | | X | | X | | X | | X | |  |
| rs1801206 | 4 | 6302707 | WFS1 | 0.59 | T | C | 0.089 | 0.013 | 1.4E-12 | 0.027 | | 0.016 | 9.23E-02 | X | | X | | X | | X | | X | |  |
| rs1801214 | 4 | 6303022 | WFS1 | 0.62 | T | C | 0.091 | 0.012 | 1.6E-13 |  | |  |  |  | |  | |  | |  | |  | |  |
| rs1046316 | 4 | 6304087 | WFS1 | 0.66 | G | A | 0.086 | 0.013 | 2.3E-11 | 0.017 | | 0.017 | 3.22E-01 | X | | X | | X | | X | | X | |  |
| rs1046317 | 4 | 6304242 | WFS1 | 0.66 | C | T | 0.086 | 0.013 | 1.9E-11 | 0.017 | | 0.018 | 3.50E-01 | X | |  | |  | |  | |  | |  |
| rs3200 | 4 | 6304878 | WFS1 | 0.53 | T | C | 0.093 | 0.012 | 3.7E-14 | 0.023 | | 0.017 | 1.64E-01 | X | | X | | X | | X | | X | |  |
| rs9986109 | 4 | 6312976 | WFS1 | 0.39 | A | G | 0.076 | 0.014 | 2.5E-08 | 0.031 | | 0.018 | 9.34E-02 | X | | X | |  | |  | |  | |  |
| rs4333261 | 4 | 6314784 | WFS1 | 0.22 | C | T | 0.092 | 0.013 | 8.5E-12 |  | |  |  |  | |  | |  | |  | |  | |  |
| rs59442809 | 4 | 6315029 | WFS1 | 0.35 | G | C | 0.093 | 0.013 | 1.2E-13 | 0.024 | | 0.017 | 1.44E-01 | X | |  | |  | |  | |  | |  |
| rs4688992 | 4 | 6316920 | WFS1 | 0.67 | G | C | 0.091 | 0.013 | 3.2E-12 | 0.007 | | 0.017 | 6.54E-01 | X | |  | |  | |  | |  | |  |
| rs2269920 | 4 | 6323454 | WFS1 | 0.35 | C | A | 0.074 | 0.014 | 6.5E-08 | 0.000 | | 0.017 | 9.99E-01 | X | |  | |  | |  | |  | |  |
| rs6446489 | 4 | 6323465 | WFS1 | 0.67 | T | C | 0.074 | 0.013 | 6.5E-09 | -0.012 | | 0.018 | 5.00E-01 | X | |  | |  | |  | |  | |  |
| rs7696558 | 4 | 6323741 | WFS1 | 0.67 | G | A | 0.075 | 0.012 | 1.7E-09 | -0.012 | | 0.017 | 4.83E-01 | X | | X | | X | | X | | X | |  |
| rs6446490 | 4 | 6324647 | WFS1 | 0.55 | A | G | 0.081 | 0.013 | 1.7E-10 | 0.003 | | 0.017 | 8.54E-01 | X | |  | |  | |  | |  | |  |
| rs4689402 | 4 | 6327669 | WFS1 | 0.63 | A | C | 0.079 | 0.013 | 4.1E-09 | 0.000 | | 0.020 | 9.95E-01 | X | |  | |  | |  | |  | |  |
| rs6446491 | 4 | 6328354 | WFS1 | 0.57 | C | G | 0.080 | 0.013 | 9.1E-10 | 0.013 | | 0.019 | 5.07E-01 | X | |  | |  | |  | |  | |  |
| rs60780116 | 4 | 185708807 | ACSL1 | 0.13 | T | C | 0.090 | 0.017 | 7.4E-08 | -0.033 | | 0.025 | 1.81E-01 | X | | X | | X | | X | | X | |  |
| rs28650790 | 5 | 55861464 | ANKRD55 | 0.18 | T | C | 0.100 | 0.016 | 7.4E-10 | 0.036 | | 0.020 | 7.31E-02 | X | | X | | X | | X | |  | |  |
| rs13199286 | 6 | 20537682 | CDKAL1 | 0.12 | T | A | 0.110 | 0.019 | 3.6E-09 | -0.004 | | 0.024 | 8.55E-01 | X | | X | | X | |  | |  | |  |
| rs9348433 | 6 | 20549801 | CDKAL1 | 0.41 | G | C | 0.100 | 0.012 | 1.7E-16 | -0.024 | | 0.016 | 1.34E-01 | X | | X | | X | | X | | X | |  |
| rs7739402 | 6 | 20558016 | CDKAL1 | 0.48 | G | A | 0.086 | 0.012 | 1.4E-12 | -0.020 | | 0.016 | 1.94E-01 | X | |  | |  | |  | |  | |  |
| rs34233572 | 6 | 20565384 | CDKAL1 | 0.10 | T | A | 0.099 | 0.017 | 1.1E-08 |  | |  |  |  | |  | |  | |  | |  | |  |
| rs1012626 | 6 | 20577561 | CDKAL1 | 0.44 | T | G | 0.098 | 0.012 | 2.3E-15 | -0.021 | | 0.016 | 1.75E-01 | X | | X | |  | |  | |  | |  |
| rs9465816 | 6 | 20582144 | CDKAL1 | 0.31 | T | G | 0.084 | 0.013 | 5.1E-11 | -0.019 | | 0.016 | 2.32E-01 | X | | X | | X | | X | | X | |  |
| rs6942313 | 6 | 20592950 | CDKAL1 | 0.31 | G | A | 0.067 | 0.013 | 6.6E-08 | -0.024 | | 0.016 | 1.26E-01 | X | |  | |  | |  | |  | |  |
| rs59633721 | 6 | 20593818 | CDKAL1 | 0.32 | G | A | 0.067 | 0.013 | 7.6E-08 | -0.020 | | 0.016 | 2.11E-01 | X | | X | | X | | X | | X | |  |
| rs67121595 | 6 | 20595092 | CDKAL1 | 0.37 | C | G | 0.091 | 0.013 | 1.4E-12 | -0.016 | | 0.016 | 3.38E-01 | X | |  | |  | |  | |  | |  |
| rs2206579 | 6 | 20625634 | CDKAL1 | 0.29 | A | G | 0.073 | 0.013 | 1.9E-08 | -0.017 | | 0.016 | 2.89E-01 | X | | X | |  | |  | |  | |  |
| rs9465850 | 6 | 20639409 | CDKAL1 | 0.20 | T | C | 0.120 | 0.016 | 3.8E-13 | 0.002 | | 0.020 | 9.03E-01 | X | |  | |  | |  | |  | |  |
| rs2876576 | 6 | 20650408 | CDKAL1 | 0.42 | A | G | 0.090 | 0.012 | 1.1E-13 | 0.003 | | 0.016 | 8.51E-01 | X | | X | | X | | X | | X | |  |
| rs9295475 | 6 | 20652765 | CDKAL1 | 0.23 | G | A | 0.140 | 0.016 | 1.8E-16 | 0.006 | | 0.020 | 7.51E-01 | X | |  | |  | |  | |  | |  |
| rs979614 | 6 | 20662123 | CDKAL1 | 0.41 | A | G | 0.091 | 0.012 | 5.3E-14 | 0.005 | | 0.016 | 7.27E-01 | X | |  | |  | |  | |  | |  |
| rs742642 | 6 | 20665081 | CDKAL1 | 0.20 | A | G | 0.190 | 0.018 | 1.4E-24 | -0.006 | | 0.023 | 7.88E-01 | X | |  | |  | |  | |  | |  |
| rs7748382 | 6 | 20665549 | CDKAL1 | 0.35 | A | G | 0.150 | 0.013 | 7.1E-30 | -0.001 | | 0.017 | 9.73E-01 | X | |  | |  | |  | |  | |  |
| rs145405564 | 6 | 20672511 | CDKAL1 | 0.02 | C | T | 0.170 | 0.021 | 7.4E-17 | 0.002 | | 0.034 | 9.51E-01 | X | | X | | X | | X | | X | |  |
| rs7451008 | 6 | 20673880 | CDKAL1 | 0.29 | C | T | 0.170 | 0.013 | 3.8E-37 | -0.010 | | 0.017 | 5.68E-01 | X | | X | | X | | X | | X | |  |
| rs1012635 | 6 | 20675295 | CDKAL1 | 0.43 | A | G | 0,095 | 0.012 | 4.4E-15 | -0.002 | | 0.016 | 8.74E-01 | X | | X | |  | |  | |  | |  |
| rs72830693 | 6 | 20683900 | CDKAL1 | 0.03 | G | C | 0.290 | 0.037 | 5.5E-15 | -0.067 | | 0.050 | 1.76E-01 | X | | X | | X | |  | |  | |  |
| rs2328548 | 6 | 20716958 | CDKAL1 | 0.27 | A | G | 0.170 | 0.016 | 2.0E-28 | -0.004 | | 0.020 | 8.42E-01 | X | | X | |  | |  | |  | |  |
| rs72832303 | 6 | 20718780 | CDKAL1 | 0.01 | G | A | 0.320 | 0.046 | 8.7E-12 |  | |  |  |  | |  | |  | |  | |  | |  |
| rs9368225 | 6 | 20721721 | CDKAL1 | 0.29 | A | G | 0.140 | 0.015 | 5.3E-21 | 0.003 | | 0.019 | 8.81E-01 | X | |  | |  | |  | |  | |  |
| rs72832308 | 6 | 20732471 | CDKAL1 | 0.13 | C | T | 0.120 | 0.018 | 5.8E-12 | -0.010 | | 0.022 | 6.63E-01 | X | |  | |  | |  | |  | |  |
| rs11753041 | 6 | 20735494 | CDKAL1 | 0.44 | C | T | 0.072 | 0.012 | 5.6E-09 | -0.018 | | 0.016 | 2.42E-01 | X | | X | |  | |  | |  | |  |
| rs10946405 | 6 | 20753441 | CDKAL1 | 0.29 | C | T | 0.079 | 0.013 | 9.7E-10 | -0.020 | | 0.016 | 2.17E-01 | X | |  | |  | |  | |  | |  |
| rs72832325 | 6 | 20759210 | CDKAL1 | 0.09 | T | G | 0.130 | 0.022 | 2.6E-09 | -0.016 | | 0.029 | 5.86E-01 | X | | X | |  | |  | |  | |  |
| rs4710946 | 6 | 20764599 | CDKAL1 | 0.58 | T | C | 0.075 | 0.012 | 3.7E-10 | -0.030 | | 0.016 | 5.67E-02 | X | | X | | X | |  | |  | |  |
| rs9350294 | 6 | 20870093 | CDKAL1 | 0.61 | C | T | 0.072 | 0.013 | 1.1E-08 | -0.007 | | 0.016 | 6.58E-01 | X | | X | | X | |  | |  | |  |
| rs4897178 | 6 | 126727908 | CENPW | 0.35 | G | T | 0.074 | 0.013 | 3.2E-09 | -0.024 | | 0.018 | 1.70E-01 | X | | X | |  | |  | |  | |  |
| rs11759026 | 6 | 126792095 | CENPW | 0.29 | G | A | 0.091 | 0.015 | 5.8E-10 | -0.022 | | 0.020 | 2.72E-01 | X | | X | | X | | X | |  | |  |
| rs1602278 | 6 | 126838605 | CENPW | 0.49 | C | A | 0.065 | 0.012 | 6.0E-08 | -0.015 | | 0.016 | 3.50E-01 | X | | X | | X | | X | |  | |  |
| rs4273712 | 6 | 126964510 |  | 0.31 | G | A | 0.075 | 0.014 | 4.0E-08 | -0.011 | | 0.017 | 5.20E-01 | X | |  | |  | |  | |  | |  |
| rs10276674 | 7 | 14922007 | DGKB | 0.22 | C | T | 0.085 | 0.016 | 5.1E-08 | -0.026 | | 0.022 | 2.30E-01 | X | | X | | X | | X | | X | |  |
| rs6967891 | 7 | 15065074 | DGKB | 0.56 | C | T | 0.069 | 0.012 | 1.4E-08 | 0.018 | | 0.016 | 2.46E-01 | X | | X | | X | | X | |  | |  |
| rs1635851 | 7 | 28187806 | JAZF1 | 0.53 | C | T | 0.069 | 0.012 | 1.5E-08 | -0.028 | | 0.016 | 8.30E-02 | X | |  | |  | |  | |  | |  |
| rs1635852 | 7 | 28189411 | JAZF1 | 0.41 | T | C | 0.092 | 0.012 | 3.0E-14 | -0.045 | | 0.016 | 4.83E-03 | X | | X | | X | | X | | X | |  |
| rs849327 | 7 | 28232457 | JAZF1 | 0.48 | A | G | 0.079 | 0.013 | 4.3E-10 | -0.039 | | 0.016 | 1.64E-02 | X | | X | |  | |  | |  | |  |
| rs3802177 | 8 | 118185025 | SLC30A8 | 0.34 | G | A | 0.110 | 0.013 | 1.7E-17 | 0.033 | | 0.017 | 5.08E-02 | X | | X | | X | | X | | X | |  |
| rs2466295 | 8 | 118185041 | SLC30A8 | 0.74 | C | T | 0.080 | 0.013 | 2.8E-10 | 0.004 | | 0.017 | 8.03E-01 | X | | X | |  | |  | |  | |  |
| rs2466294 | 8 | 118185063 | SLC30A8 | 0.62 | C | G | 0.074 | 0.012 | 2.5E-09 | 0.019 | | 0.016 | 2.19E-01 | X | | X | |  | |  | |  | |  |
| rs11774700 | 8 | 118220270 |  | 0.30 | T | C | 0.110 | 0.014 | 8.6E-15 | 0.043 | | 0.019 | 2.00E-02 | X | |  | |  | |  | |  | |  |
| rs4350011 | 8 | 118225198 |  | 0.39 | G | T | 0.092 | 0.013 | 1.6E-12 | 0.016 | | 0.017 | 3.63E-01 | X | | X | |  | |  | |  | |  |
| rs7004660 | 8 | 118233841 |  | 0.42 | A | C | 0.073 | 0.012 | 3.1E-09 | 0.001 | | 0.016 | 9.74E-01 | X | |  | |  | |  | |  | |  |
| rs997313 | 8 | 118236975 |  | 0.43 | A | T | 0.077 | 0.013 | 2.5E-09 | -0.004 | | 0.017 | 8.18E-01 | X | | X | |  | |  | |  | |  |
| rs62530366 | 8 | 145536056 | HSF1 | 0.32 | G | A | 0.076 | 0.013 | 1.9E-08 | 0.035 | | 0.020 | 7.97E-02 | X | | X | | X | | X | |  | |  |
| rs2811713 | 9 | 21999328 | CDKN2A/B | 0.18 | G | A | 0.072 | 0.012 | 5.8E-09 | 0.035 | | 0.016 | 3.12E-02 | X | | X | | X | | X | |  | |  |
| rs1101329 | 9 | 22015997 | CDKN2A/B | 0.18 | C | T | 0.078 | 0.012 | 3.0E-10 | 0.038 | | 0.016 | 1.70E-02 | X | | X | | X | | X | |  | |  |
| rs1333045 | 9 | 22119195 | CDKN2A/B | 0.51 | C | T | 0.071 | 0.012 | 1.0E-08 | 0.026 | | 0.017 | 1.24E-01 | X | | X | |  | |  | |  | |  |
| rs10757281 | 9 | 22127613 | CDKN2A/B | 0.17 | C | T | 0.140 | 0.022 | 2.3E-10 | 0.036 | | 0.032 | 2.59E-01 | X | |  | |  | |  | |  | |  |
| rs10811657 | 9 | 22127641 | CDKN2A/B | 0.17 | G | A | 0.130 | 0.019 | 1.0E-10 | 0.050 | | 0.033 | 1.30E-01 | X | | X | |  | |  | |  | |  |
| rs12379111 | 9 | 22128180 | CDKN2A/B | 0.11 | C | G | 0.140 | 0.025 | 2.7E-08 | 0.009 | | 0.033 | 7.81E-01 | X | |  | |  | |  | |  | |  |
| rs7020996 | 9 | 22129579 | CDKN2A/B | 0.20 | C | T | 0.150 | 0.020 | 4.4E-14 | -0.007 | | 0.025 | 7.69E-01 | X | |  | |  | |  | |  | |  |
| rs10965243 | 9 | 22130065 | CDKN2A/B | 0.15 | A | G | 0.160 | 0.021 | 1.4E-13 | 0.050 | | 0.026 | 5.66E-02 | X | |  | |  | |  | |  | |  |
| rs143064326 | 9 | 22132468 | CDKN2A/B | 0.03 | A | G | 0.210 | 0.036 | 8.9E-09 |  | |  |  |  | |  | |  | |  | |  | |  |
| rs72655438 | 9 | 22132897 | CDKN2A/B | 0.03 | T | G | 0.210 | 0.035 | 6.2E-09 | -0.006 | | 0.042 | 8.94E-01 | X | | X | |  | |  | |  | |  |
| rs10965250 | 9 | 22133284 | CDKN2A/B | 0.20 | G | A | 0.140 | 0.016 | 2.7E-17 | -0.007 | | 0.021 | 7.28E-01 | X | | X | | X | | X | |  | |  |
| rs12555274 | 9 | 22136440 |  | 0.27 | C | G | 0.120 | 0.014 | 1.7E-16 | -0.018 | | 0.022 | 3.94E-01 | X | | X | | X | | X | |  | |  |
| rs1333051 | 9 | 22136489 |  | 0.13 | A | T | 0.140 | 0.019 | 2.5E-12 | -0.010 | | 0.025 | 6.93E-01 | X | |  | |  | |  | |  | |  |
| rs10811663 | 9 | 22139220 |  | 0.08 | G | A | 0.140 | 0.026 | 7.5E-08 | 0.001 | | 0.043 | 9.89E-01 | X | | X | |  | |  | |  | |  |
| rs9410573 | 9 | 84311800 | TLE1 | 0.44 | T | C | 0.073 | 0.013 | 2.0E-08 | -0.004 | | 0.017 | 8.18E-01 | X | | X | | X | | X | |  | |  |
| rs10760280 | 9 | 126112812 |  | 0.38 | T | C | 0.068 | 0.013 | 7.3E-08 | 0.027 | | 0.016 | 9.17E-02 | X | | X | | X | | X | |  | |  |
| rs11257655 | 10 | 12307894 | CDC123/CAMK1D | 0.25 | T | C | 0.080 | 0.015 | 4.0E-08 | 0.004 | | 0.020 | 8.36E-01 | X | | X | | X | | X | |  | |  |
| rs703984 | 10 | 80941417 | ZMIZ1 | 0.37 | G | C | 0.079 | 0.012 | 1.5E-10 | 0.018 | | 0.016 | 2.69E-01 | X | | X | | X | | X | |  | |  |
| rs810517 | 10 | 80942620 | ZMIZ1 | 0.33 | C | T | 0.089 | 0.013 | 1.3E-12 | 0.034 | | 0.016 | 3.61E-02 | X | | X | | X | | X | |  | |  |
| rs697238 | 10 | 80947668 | ZMIZ1 | 0.61 | T | G | 0.077 | 0.013 | 8.4E-10 | 0.023 | | 0.016 | 1.48E-01 | X | |  | |  | |  | |  | |  |
| rs66503910 | 10 | 94198332 |  | 0.31 | C | T | 0.082 | 0.015 | 2.6E-08 | -0.040 | | 0.020 | 4.23E-02 | X | |  | |  | |  | |  | |  |
| rs10882063 | 10 | 94199337 |  | 0.53 | G | T | 0.077 | 0.013 | 9.7E-09 | -0.052 | | 0.019 | 5.48E-03 | X | | X | |  | |  | |  | |  |
| rs2251101 | 10 | 94211304 | HHEX/IDE | 0.74 | C | T | 0.073 | 0.014 | 9.3E-08 | -0.019 | | 0.018 | 2.97E-01 | X | | X | | X | |  | |  | |  |
| rs11187019 | 10 | 94248310 | HHEX/IDE | 0.57 | A | G | 0.099 | 0.012 | 3.2E-16 | -0.013 | | 0.016 | 4.22E-01 | X | |  | |  | |  | |  | |  |
| rs11187031 | 10 | 94261791 | HHEX/IDE | 0.19 | G | T | 0.080 | 0.014 | 6.2E-09 | -0.027 | | 0.019 | 1.52E-01 | X | | X | |  | |  | |  | |  |
| rs11187054 | 10 | 94284273 | HHEX/IDE | 0.05 | T | A | 0.085 | 0.014 | 2.4E-09 |  | |  |  |  | |  | |  | |  | |  | |  |
| rs35418143 | 10 | 94324988 | HHEX/IDE | 0.15 | A | G | 0.084 | 0.015 | 6.6E-09 | -0.013 | | 0.021 | 5.54E-01 | X | |  | |  | |  | |  | |  |
| rs6583826 | 10 | 94347830 | HHEX/IDE | 0.58 | G | A | 0.092 | 0.012 | 1.8E-14 | 0.007 | | 0.016 | 6.81E-01 | X | |  | |  | |  | |  | |  |
| rs7922041 | 10 | 94348200 | HHEX/IDE | 0.23 | G | A | 0.120 | 0.015 | 2.4E-15 | -0.030 | | 0.021 | 1.57E-01 | X | |  | |  | |  | |  | |  |
| rs7921040 | 10 | 94418425 | HHEX/IDE | 0.58 | G | A | 0.096 | 0.012 | 2.3E-15 | 0.003 | | 0.016 | 8.44E-01 | X | |  | |  | |  | |  | |  |
| rs7911264 | 10 | 94436851 | HHEX/IDE | 0.56 | T | C | 0.110 | 0.012 | 8.4E-20 | 0.015 | | 0.017 | 3.73E-01 | X | |  | |  | |  | |  | |  |
| rs10882098 | 10 | 94444793 | HHEX/IDE | 0.43 | C | T | 0.130 | 0.012 | 1.4E-26 | -0.001 | | 0.017 | 9.55E-01 | X | | X | | X | | X | | X | |  |
| rs11187133 | 10 | 94444856 | HHEX/IDE | 0.30 | G | A | 0.130 | 0.014 | 6.1E-19 | 0.004 | | 0.021 | 8.47E-01 | X | | X | |  | |  | |  | |  |
| rs146935743 | 10 | 94466064 | HHEX/IDE | 0.03 | C | T | 0.170 | 0.030 | 4.0E-08 | -0.087 | | 0.067 | 1.91E-01 | X | | X | | X | | X | | X | |  |
| rs11187140 | 10 | 94466910 | HHEX/IDE | 0.34 | G | A | 0.130 | 0.013 | 4.2E-26 | -0.008 | | 0.017 | 6.26E-01 | X | |  | |  | |  | |  | |  |
| rs61862780 | 10 | 94468643 | HHEX/IDE | 0.39 | C | T | 0.098 | 0.012 | 3.5E-16 | 0.005 | | 0.016 | 7.51E-01 | X | |  | |  | |  | |  | |  |
| rs11187146 | 10 | 94478355 | HHEX/IDE | 0.25 | G | C | 0.120 | 0.018 | 2.3E-11 | -0.006 | | 0.028 | 8.37E-01 | X | | X | |  | |  | |  | |  |
| rs185606508 | 10 | 94490348 | HHEX/IDE | 0.02 | T | G | 0.130 | 0.021 | 1.3E-09 |  | |  |  |  | |  | |  | |  | |  | |  |
| rs947591 | 10 | 94495753 | HHEX/IDE | 0.38 | A | C | 0.090 | 0.013 | 7.4E-13 | 0.002 | | 0.016 | 9.01E-01 | X | | X | |  | |  | |  | |  |
| rs2488073 | 10 | 94498975 | HHEX/IDE | 0.38 | G | A | 0.087 | 0.013 | 8.5E-12 | 0.002 | | 0.018 | 9.32E-01 | X | | X | |  | |  | |  | |  |
| rs2488071 | 10 | 94499577 | HHEX/IDE | 0.45 | G | A | 0.085 | 0.013 | 3.1E-11 | 0.003 | | 0.016 | 8.44E-01 | X | |  | |  | |  | |  | |  |
| rs113397277 | 10 | 114650787 |  | 0.03 | C | T | 0.200 | 0.035 | 1.3E-08 | -0.062 | | 0.067 | 3.57E-01 | X | | X | | X | | X | |  | |  |
| rs17746916 | 10 | 114704781 |  | 0.05 | T | C | 0.150 | 0.027 | 1.7E-08 | -0.084 | | 0.036 | 2.10E-02 | X | | X | |  | |  | |  | |  |
| rs720785 | 10 | 114721568 | TCF7L2 | 0.34 | C | G | 0.140 | 0.016 | 7.6E-19 | -0.014 | | 0.022 | 5.32E-01 | X | | X | | X | |  | |  | |  |
| rs720784 | 10 | 114721671 | TCF7L2 | 0.39 | T | A | 0.120 | 0.015 | 8.9E-15 | -0.023 | | 0.025 | 3.57E-01 | X | | X | |  | |  | |  | |  |
| rs12255678 | 10 | 114729482 | TCF7L2 | 0.18 | G | T | 0.150 | 0.015 | 2.2E-25 | -0.029 | | 0.021 | 1.64E-01 | X | |  | |  | |  | |  | |  |
| rs7901275 | 10 | 114732906 | TCF7L2 | 0.51 | C | A | 0.120 | 0.013 | 3.6E-20 | 0.013 | | 0.016 | 4.17E-01 | X | | X | | X | |  | |  | |  |
| rs12243578 | 10 | 114733456 | TCF7L2 | 0.21 | T | C | 0.180 | 0.014 | 1.4E-35 | -0.026 | | 0.020 | 1.83E-01 | X | | X | |  | |  | |  | |  |
| rs7080591 | 10 | 114740617 | TCF7L2 | 0.53 | T | C | 0.100 | 0.013 | 1.1E-15 | 0.007 | | 0.016 | 6.87E-01 | X | | X | | X | |  | |  | |  |
| rs7079711 | 10 | 114745788 | TCF7L2 | 0.16 | G | A | 0.170 | 0.018 | 8.0E-22 | 0.014 | | 0.022 | 5.18E-01 | X | |  | |  | |  | | X | |  |
| rs4073288 | 10 | 114747277 | TCF7L2 | 0.29 | A | G | 0.140 | 0.013 | 4.2E-25 | -0.014 | | 0.017 | 4.15E-01 | X | |  | |  | |  | |  | |  |
| rs11196182 | 10 | 114750157 | TCF7L2 | 0.12 | C | T | 0.150 | 0.018 | 8.1E-17 | -0.027 | | 0.023 | 2.33E-01 | X | | X | | X | |  | |  | |  |
| rs180988137 | 10 | 114751173 | TCF7L2 | 0.006 | G | A | 0.400 | 0.075 | 7.9E-08 |  | |  |  |  | |  | |  | |  | |  | |  |
| rs58064715 | 10 | 114751986 | TCF7L2 | 0.07 | A | G | 0.140 | 0.023 | 4.8E-10 | 0.072 | | 0.032 | 2.26E-02 | X | |  | |  | |  | |  | |  |
| rs116859590 | 10 | 114752410 | TCF7L2 | 0.02 | T | C | 0.290 | 0.046 | 2.9E-10 |  | |  |  |  | |  | |  | |  | |  | |  |
| rs149692182 | 10 | 114752674 | TCF7L2 | 0.02 | T | C | 0.420 | 0.076 | 2.7E-08 |  | |  |  |  | |  | |  | |  | |  | |  |
| rs35676242 | 10 | 114757314 | TCF7L2 | 0.03 | A | C | 0.210 | 0.028 | 3.8E-14 | 0.091 | | 0.038 | 1.62E-02 | X | |  | |  | |  | |  | |  |
| rs7903146 | 10 | 114758349 | TCF7L2 | 0.24 | T | C | 0.290 | 0.013 | 9.3E-108 | -0.005 | | 0.017 | 7.53E-01 | X | | X | | X | | X | | X | |  |
| rs4267006 | 10 | 114758779 | TCF7L2 | 0.17 | T | G | 0.280 | 0.015 | 4.2E-81 | -0.037 | | 0.019 | 5.28E-02 | X | |  | |  | |  | |  | |  |
| rs11196187 | 10 | 114759445 | TCF7L2 | 0.05 | A | G | 0.220 | 0.025 | 1.5E-18 | 0.077 | | 0.032 | 1.62E-02 | X | | X | | X | |  | |  | |  |
| rs61872774 | 10 | 114765390 | TCF7L2 | 0.009 | A | G | 0.360 | 0.062 | 8.0E-09 |  | |  |  |  | |  | |  | |  | |  | |  |
| rs10885404 | 10 | 114773068 | TCF7L2 | 0.18 | G | T | 0.150 | 0.018 | 6.5E-17 | -0.006 | | 0.028 | 8.32E-01 | X | | X | | X | | X | | X | |  |
| rs61872780 | 10 | 114782290 | TCF7L2 | 0.007 | A | G | 0.370 | 0.068 | 4.6E-08 |  | |  |  |  | |  | |  | |  | |  | |  |
| rs140820620 | 10 | 114787948 | TCF7L2 | 0.02 | A | G | 0.390 | 0.050 | 5.0E-15 |  | |  |  |  | |  | |  | |  | |  | |  |
| rs138732563 | 10 | 114792997 | TCF7L2 | 0.02 | C | T | 0.370 | 0.061 | 2.0E-09 |  | |  |  |  | |  | |  | |  | |  | |  |
| rs116369954 | 10 | 114793572 | TCF7L2 | 0.02 | C | T | 0.300 | 0.035 | 1.6E-17 | -0.087 | | 0.057 | 1.29E-01 | X | | X | | X | | X | | X | |  |
| rs17685538 | 10 | 114797471 | TCF7L2 | 0.07 | C | G | 0.130 | 0.023 | 2.5E-08 | 0.013 | | 0.038 | 7.37E-01 | X | | X | |  | |  | |  | |  |
| rs11196200 | 10 | 114801938 | TCF7L2 | 0.40 | G | C | 0.170 | 0.012 | 1.4E-41 | 0.005 | | 0.015 | 7.55E-01 | X | | X | |  | |  | |  | |  |
| rs61872787 | 10 | 114812960 | TCF7L2 | 0.02 | G | A | 0.360 | 0.055 | 9.0E-11 | -0.112 | | 0.106 | 2.91E-01 | X | | X | | X | | X | | X | |  |
| rs7071302 | 10 | 114817527 | TCF7L2 | 0.39 | G | T | 0.160 | 0.012 | 2.3E-38 | 0.006 | | 0.017 | 7.32E-01 | X | | X | | X | | X | | X | |  |
| rs35519679 | 10 | 114818754 | TCF7L2 | 0.23 | A | G | 0,220 | 0,015 | 1,4E-50 | 0.016 | | 0.019 | 4.08E-01 | X | | X | |  | |  | |  | |  |
| rs35936842 | 10 | 114818772 | TCF7L2 | 0.32 | A | G | 0,100 | 0,013 | 1,3E-16 | 0.020 | | 0.017 | 2.34E-01 | X | | X | | X | | X | | X | |  |
| rs12245680 | 10 | 114820191 | TCF7L2 | 0.08 | C | T | 0,140 | 0,021 | 1,1E-10 | 0.038 | | 0.028 | 1.81E-01 | X | | X | |  | |  | |  | |  |
| rs61872790 | 10 | 114821527 | TCF7L2 | 0.12 | G | A | 0,230 | 0,018 | 4,6E-37 | -0.016 | | 0.023 | 4.96E-01 | X | | X | |  | |  | |  | |  |
| rs11196213 | 10 | 114821554 | TCF7L2 | 0.38 | T | C | 0,088 | 0,012 | 7,3E-13 | 0.012 | | 0.016 | 4.51E-01 | X | | X | |  | |  | |  | |  |
| rs7070182 | 10 | 114822698 | TCF7L2 | 0.14 | T | C | 0,091 | 0,017 | 6,7E-08 | -0.005 | | 0.022 | 8.30E-01 | X | | X | | X | |  | |  | |  |
| rs10885410 | 10 | 114824473 | TCF7L2 | 0.21 | G | A | 0,100 | 0,015 | 1,6E-11 | 0.024 | | 0.019 | 2.03E-01 | X | | X | | X | |  | |  | |  |
| rs4918791 | 10 | 114830306 | TCF7L2 | 0.39 | G | A | 0,094 | 0,016 | 2,4E-09 |  | |  |  |  | |  | |  | |  | |  | |  |
| rs116929578 | 10 | 114836181 | TCF7L2 | 0.03 | G | A | 0,230 | 0,035 | 1,4E-10 |  | |  |  |  | |  | |  | |  | |  | |  |
| rs7069881 | 10 | 114838872 | TCF7L2 | 0.31 | C | T | 0,076 | 0,013 | 1,4E-08 | 0.007 | | 0.018 | 7.15E-01 | X | | X | |  | |  | |  | |  |
| rs12259231 | 10 | 114840745 | TCF7L2 | 0.17 | C | G | 0.100 | 0.016 | 2.2E-10 | 0.035 | | 0.022 | 1.03E-01 | X | | X | | X | | X | | X | |  |
| rs190497613 | 10 | 114844669 | TCF7L2 | 0.12 | C | A | 0.093 | 0.017 | 6.0E-08 |  | |  |  |  | |  | |  | |  | |  | |  |
| rs10885414 | 10 | 114861304 | TCF7L2 | 0.24 | A | G | 0.081 | 0.014 | 5.8E-09 | 0.037 | | 0.019 | 5.57E-02 | X | |  | |  | |  | |  | |  |
| rs11196229 | 10 | 114866172 |  | 0.20 | G | A | 0.086 | 0.015 | 5.2E-09 | 0.034 | | 0.019 | 7.57E-02 | X | |  | |  | |  | |  | |  |
| rs7080960 | 10 | 124122632 |  | 0.54 | T | C | 0.067 | 0.012 | 2.3E-08 | 0.059 | | 0.016 | 2.15E-04 | X | | X | |  | |  | |  | |  |
| rs35777422 | 10 | 124180176 | PLEKHA1 | 0.32 | G | A | 0.070 | 0.013 | 6.4E-08 | 0.054 | | 0.016 | 9.15E-04 | X | |  | |  | |  | |  | |  |
| rs45519541 | 10 | 124183691 | PLEKHA1 | 0.09 | T | C | 0.083 | 0.015 | 8.1E-08 |  | |  |  |  | |  | |  | |  | |  | |  |
| rs2292626 | 10 | 124186714 | PLEKHA1 | 0.50 | C | T | 0.085 | 0.012 | 1.8E-12 | 0.049 | | 0.015 | 1.38E-03 | X | | X | | X | | X | |  | |  |
| rs1045216 | 10 | 124189197 | PLEKHA1 | 0.64 | A | G | 0.069 | 0.012 | 2.1E-08 | 0.032 | | 0.016 | 3.91E-02 | X | |  | |  | |  | |  | |  |
| rs756852 | 11 | 2663891 | KCNQ1 | 0.28 | G | A | 0.090 | 0.014 | 3.6E-10 | -0.007 | | 0.019 | 6.94E-01 | X | | X | | X | | X | |  | |  |
| rs231362 | 11 | 2691471 | KCNQ1 | 0.63 | G | A | 0.077 | 0.013 | 1.9E-09 | 0.030 | | 0.017 | 7.37E-02 | X | | X | |  | |  | |  | |  |
| rs231360 | 11 | 2692249 | KCNQ1 | 0.45 | T | C | 0.079 | 0.013 | 9.5E-10 | 0.035 | | 0.017 | 4.22E-02 | X | | X | | X | | X | | X | |  |
| rs233449 | 11 | 2843803 | KCNQ1 | 0.24 | G | A | 0.089 | 0.014 | 2.8E-10 | 0.008 | | 0.018 | 6.40E-01 | X | | X | | X | |  | |  | |  |
| rs163184 | 11 | 2847069 | KCNQ1 | 0.43 | G | T | 0.090 | 0.012 | 3.4E-13 | 0.019 | | 0.016 | 2.43E-01 | X | |  | |  | |  | |  | |  |
| rs2283228 | 11 | 2849530 | KCNQ1 | 0.14 | A | C | 0.150 | 0.025 | 4.1E-09 | -0.009 | | 0.033 | 7.76E-01 | X | | X | |  | |  | |  | |  |
| rs234858 | 11 | 2852857 | KCNQ1 | 0.21 | G | A | 0.091 | 0.014 | 3.1E-10 | 0.012 | | 0.018 | 5.04E-01 | X | | X | | X | | X | | X | |  |
| rs2237895 | 11 | 2857194 | KCNQ1 | 0.35 | C | A | 0.097 | 0.013 | 1.7E-13 | 0.006 | | 0.018 | 7.62E-01 | X | | X | | X | | X | | X | |  |
| rs60808706 | 11 | 2857233 | KCNQ1 | 0.11 | G | A | 0.150 | 0.028 | 8.8E-08 | -0.026 | | 0.049 | 5.97E-01 | X | |  | |  | |  | |  | |  |
| rs2237897 | 11 | 2858546 | KCNQ1 | 0.12 | C | T | 0.220 | 0.031 | 4.9E-13 | -0.038 | | 0.043 | 3.71E-01 | X | | X | | X | | X | | X | |  |
| rs5213 | 11 | 17408404 |  | 0.66 | C | T | 0.068 | 0.013 | 4.9E-08 | 0.028 | | 0.016 | 8.11E-02 | X | | X | | X | | X | |  | |  |
| rs5219 | 11 | 17409572 | KCNJ11 | 0.62 | T | C | 0.068 | 0.012 | 4.3E-08 | 0.028 | | 0.016 | 8.05E-02 | X | | X | | X | | X | | X | |  |
| rs7947967 | 11 | 43854610 | HSD17B12 | 0.52 | G | C | 0.074 | 0.013 | 1.4E-08 |  | |  |  |  | |  | |  | |  | |  | |  |
| rs1061810 | 11 | 43877934 | HSD17B12 | 0.31 | A | C | 0.080 | 0.014 | 5.3E-09 | 0.010 | | 0.017 | 5.58E-01 | X | | X | | X | | X | |  | |  |
| rs35186585 | 11 | 43878534 | HSD17B12 | 0.22 | C | A | 0.083 | 0.015 | 2.2E-08 | 0.009 | | 0.019 | 6.51E-01 | X | |  | |  | |  | |  | |  |
| rs76550717 | 11 | 72428172 | ARAP1 | 0.13 | A | G | 0.096 | 0.016 | 3.8E-09 | 0.002 | | 0.022 | 9.41E-01 | X | | X | | X | | X | | X | |  |
| rs1783598 | 11 | 72851463 |  | 0.78 | T | C | 0.084 | 0.015 | 4.1E-08 | -0.018 | | 0.021 | 3.79E-01 | X | |  | |  | |  | |  | |  |
| rs4238013 | 12 | 4376089 | CCND2 | 0.65 | C | T | 0.099 | 0.017 | 3.6E-09 | 0.033 | | 0.027 | 2.22E-01 | X | | X | | X | | X | |  | |  |
| rs66947454 | 12 | 121350922 |  | 0.13 | T | C | 0.081 | 0.015 | 4.6E-08 | 0.013 | | 0.024 | 5.82E-01 | X | | X | |  | |  | |  | |  |
| rs11065397 | 12 | 121451910 | HNF1A (TCF1) | 0.25 | C | T | 0.072 | 0.013 | 3.6E-08 | -0.004 | | 0.017 | 7.98E-01 | X | | X | | X | | X | | X | |  |
| rs7131696 | 12 | 121459344 |  | 0.14 | T | C | 0.080 | 0.015 | 4.6E-08 | 0.002 | | 0.020 | 9.30E-01 | X | |  | |  | |  | |  | |  |
| rs11616380 | 13 | 80705315 | SPRY2 | 0.27 | G | T | 0.090 | 0.014 | 3.9E-11 | 0.007 | | 0.017 | 6.93E-01 | X | | X | | X | | X | |  | |  |
| rs7998259 | 13 | 80718654 | SPRY2 | 0.31 | G | A | 0.082 | 0.014 | 2.4E-09 | -0.010 | | 0.018 | 5.66E-01 | X | | X | |  | |  | |  | |  |
| rs4774420 | 15 | 62117975 | C2CD4A | 0.31 | C | T | 0.075 | 0.013 | 2.7E-08 | 0.013 | | 0.018 | 4.72E-01 | X | | X | | X | | X | |  | |  |
| rs11856307 | 15 | 62399093 | C2CD4A | 0.39 | A | C | 0.066 | 0.012 | 8.0E-08 | -0.006 | | 0.016 | 6.95E-01 | X | | X | | X | |  | |  | |  |
| rs11072653 | 15 | 77693934 |  | 0.65 | T | C | 0.075 | 0.014 | 5.5E-08 | -0.014 | | 0.018 | 4.31E-01 | X | |  | |  | |  | |  | |  |
| rs952471 | 15 | 77776498 | HMG20A | 0.58 | G | C | 0.082 | 0.013 | 4.0E-10 | -0.013 | | 0.017 | 4.30E-01 | X | |  | |  | |  | |  | |  |
| rs4784323 | 16 | 53797565 | FTO | 0.70 | G | A | 0.077 | 0.014 | 5.3E-08 | 0.017 | | 0.020 | 3.92E-01 | X | | X | |  | |  | |  | |  |
| rs7206790 | 16 | 53797908 | FTO | 0.42 | G | C | 0.110 | 0.013 | 7.6E-19 | 0.004 | | 0.017 | 8.29E-01 | X | |  | |  | |  | |  | |  |
| rs8047395 | 16 | 53798523 | FTO | 0.54 | A | G | 0.100 | 0.012 | 3.2E-16 | 0.005 | | 0.016 | 7.41E-01 | X | |  | |  | |  | |  | |  |
| rs9930333 | 16 | 53799977 | FTO | 0.38 | G | T | 0.120 | 0.012 | 5.2E-24 | -0.002 | | 0.016 | 9.16E-01 | X | | X | | X | | X | | X | |  |
| rs1558902 | 16 | 53803574 | FTO | 0.32 | A | T | 0.130 | 0.012 | 4.7E-25 | 0.003 | | 0.016 | 8.51E-01 | X | | X | | X | | X | | X | |  |
| rs2058908 | 16 | 53806145 | FTO | 0.74 | C | T | 0.077 | 0.014 | 6.1E-08 | 0.045 | | 0.021 | 2.95E-02 | X | |  | |  | |  | |  | |  |
| rs4783819 | 16 | 53816647 | FTO | 0.65 | C | G | 0.076 | 0.013 | 1.6E-09 | 0.034 | | 0.016 | 3.63E-02 | X | |  | |  | |  | |  | |  |
| rs113191842 | 16 | 53817318 | FTO | 0.08 | A | G | 0.160 | 0.023 | 8.5E-12 |  | |  |  |  | |  | |  | |  | |  | |  |
| rs9927317 | 16 | 53820996 | FTO | 0.31 | G | C | 0.120 | 0.012 | 7.1E-23 | 0.006 | | 0.016 | 6.88E-01 | X | | X | | X | | X | | X | |  |
| rs28432761 | 16 | 53823878 | FTO | 0.16 | C | T | 0.098 | 0.012 | 1.4E-15 | 0.026 | | 0.016 | 1.02E-01 | X | |  | |  | |  | |  | |  |
| rs1861867 | 16 | 53848561 | FTO | 0.64 | G | A | 0.097 | 0.014 | 5.5E-12 | 0.034 | | 0.020 | 9.26E-02 | X | |  | |  | |  | |  | |  |
| rs8056223 | 16 | 75233867 | BCAR1 | 0.08 | T | G | 0.180 | 0.033 | 3.7E-08 | -0.044 | | 0.058 | 4.44E-01 | X | | X | |  | |  | |  | |  |
| rs889512 | 16 | 75242012 | BCAR1 | 0.09 | C | G | 0.120 | 0.021 | 7.5E-09 | -0.029 | | 0.025 | 2.37E-01 | X | |  | |  | |  | |  | |  |
| rs8056814 | 16 | 75252327 | BCAR1 | 0.12 | G | A | 0.150 | 0.023 | 3.7E-11 | -0.041 | | 0.029 | 1.57E-01 | X | | X | | X | | X | |  | |  |
| rs150037540 | 16 | 75255320 | BCAR1 | 0.02 | C | T | 0.220 | 0.041 | 9.5E-08 | -0.058 | | 0.053 | 2.76E-01 | X | |  | |  | |  | |  | |  |
| rs2925979 | 16 | 81534790 | CMIP | 0.71 | T | C | 0.074 | 0.013 | 2.7E-08 | 0.006 | | 0.018 | 7.20E-01 | X | | X | | X | | X | | X | |  |
| rs78761021 | 17 | 9780387 | GLP2R | 0.22 | G | A | 0.070 | 0.013 | 5.5E-08 | -0.031 | | 0.017 | 7.24E-02 | X | | X | | X | | X | | X | |  |
| rs17743194 | 17 | 9788251 |  | 0.20 | A | G | 0.071 | 0.013 | 8.0E-08 | -0.030 | | 0.017 | 7.83E-02 | X | | X | | X | | X | |  | |  |
| - | 17 | 36101633 | HNF1B (TCF2) | 0.40 | G | T | 0.081 | 0.013 | 2.0E-09 | -0.004 | | 0.018 | 8.40E-01 | X | | X | | X | | X | |  | |  |
| rs757209 | 17 | 36102833 | HNF1B (TCF2) | 0.40 | G | A | 0.083 | 0.014 | 1.1E-09 | -0.005 | | 0.018 | 8.01E-01 | X | | X | | X | | X | |  | |  |
| rs11263763 | 17 | 36103565 | HNF1B (TCF2) | 0.58 | G | A | 0.078 | 0.013 | 5.5E-09 | 0.005 | | 0.018 | 7.75E-01 | X | |  | |  | |  | |  | |  |
| rs6857 | 19 | 45392254 |  | 0.13 | C | T | 0.099 | 0.018 | 5.6E-08 | -1.161 | | 0.023 | 0.00E+00 |  | |  | |  | |  | |  | |  |
| rs769449 | 19 | 45410002 | APOE | 0.12 | G | A | 0.110 | 0.021 | 6.0E-08 | -1.259 | | 0.026 | 0.00E+00 |  | |  | |  | |  | |  | |  |
| rs429358 | 19 | 45411941 | APOE | 0.09 | T | C | 0.120 | 0.019 | 1.4E-10 | -1.350 | | 0.027 | 0.00E+00 |  | |  | |  | |  | |  | |  |
| rs4420638 | 19 | 45422946 |  | 0.16 | A | G | 0.110 | 0.018 | 1.5E-09 | -1.351 | | 0.032 | 0.00E+00 |  | |  | |  | |  | |  | |  |
| rs1548684 | 22 | 30138232 |  | 0.06 | G | A | 0.120 | 0.022 | 8.4E-08 | -0.049 | | 0.027 | 7.45E-02 | X | |  | |  | |  | |  | |  |
| rs148945987 | 22 | 30140375 |  | 0.01 | C | T | 0.120 | 0.022 | 7.4E-08 |  | |  |  |  | |  | |  | |  | |  | |  |
| rs73883363 | 22 | 30458393 | MTMR3/HORMAD2 | 0.06 | T | A | 0.120 | 0.022 | 8.9E-08 | -0.042 | | 0.028 | 1.29E-01 | X | | X | | X | | X | |  | |  |
| rs148685805 | 22 | 30484095 | MTMR3/HORMAD2 | 0.001 | C | T | 0.140 | 0.026 | 8.1E-08 | -0.057 | | 0.049 | 2.45E-01 | X | | X | | X | | X | |  | |  |
| rs146995062 | 22 | 30507019 | MTMR3/HORMAD2 | 0.06 | C | G | 0.120 | 0.022 | 3.6E-08 | -0.042 | | 0.028 | 1.31E-01 | X | | X | | X | | X | |  | |  |

Information on genetic variants used in the two-sample Mendelian randomization study on type 2 diabetes from the DIAGRAM consortium and risk of Alzheimer’s disease from the IGAP consortium. The five most right columns identify which variants were included in the five genetic instruments (marked by X). MAF=Minor Allele Frequency; LD=Linkage Disequilibrium; SNP=Single Nucleotide Polymorphism.

**Supplementary Table 2: Stratified Cox sensitivity analysis to check proportional hazards assumption.**

| **Alzheimer's disease** |  |  |  |  |  |  |  |  |  |  |  |  |  |  |  |  |  |  |
| --- | --- | --- | --- | --- | --- | --- | --- | --- | --- | --- | --- | --- | --- | --- | --- | --- | --- | --- |
|  | **Hazard ratio compared to reference** | | | |  |  |  |  |  |  |  |  |  |  |  |  |  |  |
| **Sex adjusted** | T2D (1) | Sex (female) |  |  |  |  |  |  |  |  |  |  |  |  |  |  |  |  |
| Fully adjusted | 1.15 | 1.14 |  |  |  |  |  |  |  |  |  |  |  |  |  |  |  |  |
| Type 2 diabetes stratified |  | 1.14 |  |  |  |  |  |  |  |  |  |  |  |  |  |  |  |  |
| Sex stratified | 1.15 |  |  |  |  |  |  |  |  |  |  |  |  |  |  |  |  |  |
|  |  |  |  |  |  |  |  |  |  |  |  |  |  |  |  |  |  |  |
|  | **Hazard ratio compared to reference** | | | | | | | | | | | | | | | | | |
| **Multifactorial adjusted** | T2D (1) | Sex (female) | Civil status (1) | Civil status (2) | Civil status (3) | Education group (2) | Education group (3) | Education group (4) | Education group (8) | Education group (9) | Size of community (2) | Size of community (3) | Size of community (4) | Region (2) | Region (3) | Region (4) | Region (5) |  |
| Fully adjusted | 1.13 | 1.11 | 0.81 | 0.97 | 1.00 | 1.02 | 0.95 | 0.98 | 0.82 | 0.69 | 1.41 | 0.78 | 0.63 | 0.63 | 1.10 | 0.94 | 0.52 |  |
| Type 2 diabetes stratified |  | 1.11 | 0.80 | 0.97 | 1.00 | 1.02 | 0.95 | 0.98 | 0.82 | 0.69 | 1.41 | 0.78 | 0.63 | 0.63 | 1.10 | 0.94 | 0.52 |  |
| Sex stratified | 1.13 |  | 0.80 | 0.97 | 1.00 | 1.02 | 0.95 | 0.98 | 0.82 | 0.69 | 1.41 | 0.78 | 0.63 | 0.63 | 1.10 | 0.94 | 0.52 |  |
| Civil status stratified | 1.13 | 1.11 |  |  |  | 1.02 | 0.95 | 0.98 | 0.82 | 0.69 | 1.41 | 0.78 | 0.63 | 0.63 | 1.10 | 0.94 | 0.52 |  |
| Education stratified | 1.13 | 1.11 | 0.80 | 0.97 | 1.00 |  |  |  |  |  | 1.41 | 0.78 | 0.63 | 0.63 | 1.10 | 0.94 | 0.52 |  |
| Size of community stratified | 1.13 | 1.11 | 0.81 | 0.97 | 1.00 | 1.02 | 0.95 | 0.98 | 0.82 | 0.70 |  |  |  | 0.63 | 1.10 | 0.94 | 0.52 |  |
| Region stratified | 1.13 | 1.11 | 0.81 | 0.97 | 1.00 | 1.02 | 0.95 | 0.98 | 0.82 | 0.70 | 1.41 | 0.78 | 0.63 |  |  |  |  |  |
| Ethnicity stratified | 1.13 | 1.11 | 0.81 | 0.97 | 1.00 | 1.02 | 0.95 | 0.98 | 0.82 | 0.70 | 1.41 | 0.78 | 0.63 | 0.63 | 1.10 | 0.94 | 0.52 |  |
| Split-category stratified | 1.13 | 1.11 | 0.81 | 0.97 | 1.00 | 1.02 | 0.96 | 0.98 | 0.84 | 0.70 | 1.40 | 0.78 | 0.63 | 0.63 | 1.11 | 0.94 | 0.52 |  |

| **Vascular dementia** |  |  |  |  |  |  |  |  |  |  |  |  |  |  |  |  |  |
| --- | --- | --- | --- | --- | --- | --- | --- | --- | --- | --- | --- | --- | --- | --- | --- | --- | --- |
|  | **Hazard ratio compared to reference** | | | |  |  |  |  |  |  |  |  |  |  |  |  |  |
| **Sex adjusted** | T2D (1) | Sex (female) |  |  |  |  |  |  |  |  |  |  |  |  |  |  |  |
| Fully adjusted | 2.01 | 0.81 |  |  |  |  |  |  |  |  |  |  |  |  |  |  |  |
| Type 2 diabetes stratified |  | 0.81 |  |  |  |  |  |  |  |  |  |  |  |  |  |  |  |
| Sex stratified | 2.01 |  |  |  |  |  |  |  |  |  |  |  |  |  |  |  |  |
|  |  |  |  |  |  |  |  |  |  |  |  |  |  |  |  |  |  |
|  | **Hazard ratio compared to reference** | | | | | | | | | | | | | | | | |
| **Multifactorial adjusted** | T2D (1) | Sex (female) | Civil status (1) | Civil status (2) | Civil status (3) | Education group (2) | Education group (3) | Education group (4) | Education group (8) | Education group (9) | Size of community (2) | Size of community (3) | Size of community (4) | Region (2) | Region (3) | Region (4) | Region (5) |
| Fully adjusted | 1.98 | 0.76 | 1.02 | 1.06 | 1.27 | 0.94 | 0.81 | 0.80 | 0.87 | 0.77 | 1.27 | 0.81 | 0.71 | 0.71 | 0.74 | 1.06 | 0.71 |
| Type 2 diabetes stratified |  | 0.76 | 1.01 | 1.06 | 1.27 | 0.94 | 0.82 | 0.81 | 0.87 | 0.76 | 1.27 | 0.81 | 0.71 | 0.71 | 0.74 | 1.06 | 0.71 |
| Sex stratified | 1.97 |  | 1.01 | 1.05 | 1.26 | 0.93 | 0.81 | 0.80 | 0.87 | 0.76 | 1.27 | 0.81 | 0.71 | 0.71 | 0.74 | 1.06 | 0.71 |
| Civil status stratified | 1.98 | 0.76 |  |  |  | 0.94 | 0.82 | 0.80 | 0.88 | 0.76 | 1.27 | 0.81 | 0.71 | 0.71 | 0.74 | 1.06 | 0.71 |
| Education stratified | 1.98 | 0.76 | 1.02 | 1.06 | 1.27 |  |  |  |  |  | 1.27 | 0.81 | 0.71 | 0.71 | 0.74 | 1.06 | 0.71 |
| Size of community stratified | 1.98 | 0.76 | 1.02 | 1.06 | 1.27 | 0.94 | 0.82 | 0.81 | 0.87 | 0.77 |  |  |  | 0.71 | 0.74 | 1.06 | 0.71 |
| Region stratified | 1.98 | 0.76 | 1.02 | 1.06 | 1.27 | 0.94 | 0.82 | 0.81 | 0.87 | 0.77 | 1.28 | 0.81 | 0.71 |  |  |  |  |
| Ethnicity stratified | 1.98 | 0.76 | 1.02 | 1.06 | 1.27 | 0.94 | 0.82 | 0.80 | 0.87 | 0.78 | 1.27 | 0.81 | 0.71 | 0.71 | 0.74 | 1.06 | 0.71 |
| Split-category stratified | 1.98 | 0.76 | 1.02 | 1.06 | 1.27 | 0.94 | 0.82 | 0.80 | 0.80 | 0.77 | 1.26 | 0.81 | 0.71 | 0.71 | 0.74 | 1.06 | 0.71 |

| **Unspecified dementia** | |  |  |  |  |  |  |  |  |  |  |  |  |  |  |  |  |
| --- | --- | --- | --- | --- | --- | --- | --- | --- | --- | --- | --- | --- | --- | --- | --- | --- | --- |
|  | **Hazard ratio compared to reference** | | | |  |  |  |  |  |  |  |  |  |  |  |  |  |
| **Sex adjusted** | T2D (1) | Sex (female) |  |  |  |  |  |  |  |  |  |  |  |  |  |  |  |
| Fully adjusted | 1.57 | 1.02 |  |  |  |  |  |  |  |  |  |  |  |  |  |  |  |
| Type 2 diabetes stratified |  | 1.02 |  |  |  |  |  |  |  |  |  |  |  |  |  |  |  |
| Sex stratified | 1.57 |  |  |  |  |  |  |  |  |  |  |  |  |  |  |  |  |
|  |  |  |  |  |  |  |  |  |  |  |  |  |  |  |  |  |  |
|  | **Hazard ratio compared to reference** | | | | | | | | | | | | | | | | |
| **Multifactorial adjusted** | T2D (1) | Sex (female) | Civil status (1) | Civil status (2) | Civil status (3) | Education group (2) | Education group (3) | Education group (4) | Education group (8) | Education group (9) | Size of community (2) | Size of community (3) | Size of community (4) | Region (2) | Region (3) | Region (4) | Region (5) |
| Fully adjusted | 1.53 | 0.93 | 1.21 | 1.11 | 1.35 | 0.91 | 0.81 | 0.77 | 0.93 | 0.76 | 1.24 | 0.80 | 0.67 | 0.62 | 0.84 | 1.04 | 0.68 |
| Type 2 diabetes stratified |  | 0.93 | 1.21 | 1.11 | 1.35 | 0.92 | 0.81 | 0.77 | 0.94 | 0.76 | 1.24 | 0.80 | 0.67 | 0.62 | 0.84 | 1.04 | 0.68 |
| Sex stratified | 1.53 |  | 1.21 | 1.1 | 1.35 | 0.91 | 0.81 | 0.77 | 0.93 | 0.76 | 1.24 | 0.80 | 0.67 | 0.62 | 0.84 | 1.04 | 0.68 |
| Civil status stratified | 1.53 | 0.94 |  |  |  | 0.92 | 0.81 | 0.77 | 0.94 | 0.76 | 1.24 | 0.80 | 0.67 | 0.62 | 0.84 | 1.04 | 0.68 |
| Education stratified | 1.53 | 0.93 | 1.21 | 1.11 | 1.35 |  |  |  |  |  | 1.24 | 0.80 | 0.67 | 0.62 | 0.84 | 1.04 | 0.68 |
| Size of community stratified | 1.53 | 0.93 | 1.21 | 1.11 | 1.35 | 0.91 | 0.81 | 0.77 | 0.93 | 0.77 |  |  |  | 0.62 | 0.84 | 1.04 | 0.68 |
| Region stratified | 1.53 | 0.93 | 1.21 | 1.11 | 1.35 | 0.92 | 0.81 | 0.78 | 0.93 | 0.77 | 1.24 | 0.80 | 0.67 |  |  |  |  |
| Ethnicity stratified | 1.53 | 0.93 | 1.21 | 1.11 | 1.35 | 0.91 | 0.81 | 0.77 | 0.93 | 0.76 | 1.24 | 0.80 | 0.67 | 0.62 | 0.84 | 1.04 | 0.68 |
| Splitcategory stratified | 1.53 | 0.93 | 1.21 | 1.11 | 1.35 | 0.92 | 0.81 | 0.78 | 1.03 | 0.76 | 1.24 | 0.80 | 0.67 | 0.62 | 0.84 | 1.04 | 0.68 |

| **All cause dementia** |  |  |  |  |  |  |  |  |  |  |  |  |  |  |  |  |  | |
| --- | --- | --- | --- | --- | --- | --- | --- | --- | --- | --- | --- | --- | --- | --- | --- | --- | --- | --- |
|  | **Hazard ratio compared to reference** | | | |  |  |  |  |  |  |  |  |  |  |  |  |  | |
| **Sex adjusted** | T2D (1) | Sex (female) |  |  |  |  |  |  |  |  |  |  |  |  |  |  |  | |
| Fully adjusted | 1.51 | 1.03 |  |  |  |  |  |  |  |  |  |  |  |  |  |  |  | |
| Type 2 diabetes stratified |  | 1.03 |  |  |  |  |  |  |  |  |  |  |  |  |  |  |  | |
| Sex stratified | 1.51 |  |  |  |  |  |  |  |  |  |  |  |  |  |  |  |  | |
|  |  |  |  |  |  |  |  |  |  |  |  |  |  |  |  |  |  | |
|  | **Hazard ratio compared to reference** | | | | | | | | | | | | | | | | |  |
| **Multifactorial adjusted** | T2D (1) | Sex (female) | Civil status (1) | Civil status (2) | Civil status (3) | Education group (2) | Education group (3) | Education group (4) | Education group (8) | Education group (9) | Size of community (2) | Size of community (3) | Size of community (4) | Region (2) | Region (3) | Region (4) | Region (5) | |
| Fully adjusted | 1.48 | 0.96 | 1.15 | 1.07 | 1.28 | 0.93 | 0.84 | 0.81 | 0.91 | 0.77 | 1.16 | 0.80 | 0.65 | 0.65 | 0.87 | 1.03 | 0.67 | |
| Type 2 diabetes stratified |  | 0.96 | 1.15 | 1.07 | 1.28 | 0.93 | 0.84 | 0.82 | 0.91 | 0.77 | 1.16 | 0.80 | 0.65 | 0.65 | 0.88 | 1.03 | 0.67 | |
| Sex stratified | 1.48 |  | 1.15 | 1.07 | 1.28 | 0.93 | 0.84 | 0.82 | 0.91 | 0.77 | 1.16 | 0.80 | 0.65 | 0.65 | 0.88 | 1.03 | 0.67 | |
| Civil status stratified | 1.48 | 0.96 |  |  |  | 0.94 | 0.85 | 0.82 | 0.92 | 0.77 | 1.16 | 0.80 | 0.65 | 0.65 | 0.88 | 1.03 | 0.67 | |
| Education stratified | 1.48 | 0.96 | 1.15 | 1.07 | 1.28 |  |  |  |  |  | 1.16 | 0.80 | 0.65 | 0.65 | 0.88 | 1.03 | 0.67 | |
| Size community stratified | 1.48 | 0.96 | 1.15 | 1.07 | 1.28 | 0.93 | 0.84 | 0.82 | 0.91 | 0.77 |  |  |  | 0.65 | 0.88 | 1.03 | 0.67 | |
| Region stratified | 1.48 | 0.96 | 1.15 | 1.07 | 1.29 | 0.94 | 0.85 | 0.83 | 0.91 | 0.78 | 1.16 | 0.80 | 0.65 |  |  |  |  | |
| Ethnicity stratified | 1.48 | 0.96 | 1.15 | 1.07 | 1.28 | 0.93 | 0.84 | 0.82 | 0.91 | 0.76 | 1.16 | 0.80 | 0.65 | 0.65 | 0.88 | 1.03 | 0.67 | |
| Splitcategory stratified | 1.48 | 0.96 | 1.15 | 1.07 | 1.28 | 0.94 | 0.85 | 0.82 | 0.98 | 0.77 | 1.16 | 0.80 | 0.65 | 0.65 | 0.88 | 1.03 | 0.67 | |

Hazard ratio estimates when stratifying individually on covariates. Here stratifying means that a separate baseline hazard is estimated for each strata. This is used to check the proportional hazards assumption. No major violations were found. TD2: Type 2 diabetes.

**Supplementary Figure 1: MR-Egger plot with *APOE* SNPs.**

Scatterplot of gene-outcome versus gene-exposure associations including four variants in or in linkage disequilibrium with *APOE* (lower left part of the plot). The LD<0.8 instrument was used.

**Supplementary Figure 2: MR-Egger sensitivity analysis.**

Causal risk, pleiotropy and NOME estimates for each of the genetic instruments for MR-Egger regression and SIMEX simulation.

**Supplementary Figure 3: Funnel and MR-Egger plots for the five genetic instruments.**

| **Funnel plot** | **MR-Egger plot** |
| --- | --- |
| **Pathway LD<0.2**   |  |
| **LD<0.2**   |  |
| **LD<0.4**  **** |  |

| **LD<0.6**  **** |  |  |
| --- | --- | --- |
| **LD<0.8**  **** |  | |

Funnel plots and MR-Egger plots of the five genetic instruments. Funnel plots are the genetic variants individually plotted as causal effect (βiv) versus instrument strength. Dashed line: Inverse variance weighted estimate. Long dashed line: MR-Egger estimate. MR-Egger plots are diabetes associations versus Alzheimer’s disease association (β-coefficients) for each individual variant.

**Supplementary Figure 4: Forrest plot of individual variants in pathway instrument.**

Forrest plot of the individual variants comprising the pathway instrument. To the left variant id (SNP id) and the associated gene is shown. To the right the causal odds ratio for the individual variant is shown. Estimates for the inverse variance weighted and MR-Egger regressions are shown at the bottom. Variants with a red box have estimated F-statistics above 100.

**Supplementary Figure 5: Causal estimate dependence on strength of instrument.**

a)

b)

A) Causal and observational risk estimates of Alzheimer’s disease for type 2 diabetes for pathway genetic instrument and pathway genetic instruments restricted to variants with individual estimated F-statistics above 50, 75 and 100, respectively. LD=Linkage disequilibrium; SNP=Single nucleotide polymorphism.

B) Causal risk, pleiotropy and NO Measurement Error (NOME) estimates for the pathway instruments restricted to variants with F-statistics above 50, 75, and 100, respectively.
